# The impact of almonds and almond processing on gastrointestinal physiology, luminal microbiology, and gastrointestinal symptoms: a randomized controlled trial and mastication study

**DOI:** 10.1093/ajcn/nqac265

**Published:** 2022-09-20

**Authors:** Alice C Creedon, Eirini Dimidi, Estella S Hung, Megan Rossi, Christopher Probert, Terri Grassby, Jesus Miguens-Blanco, Julian R Marchesi, S Mark Scott, Sarah E Berry, Kevin Whelan

**Affiliations:** Department of Nutritional Sciences, King's College London, London, United Kingdom; Department of Nutritional Sciences, King's College London, London, United Kingdom; Department of Nutritional Sciences, King's College London, London, United Kingdom; Department of Nutritional Sciences, King's College London, London, United Kingdom; Department of Molecular and Clinical Cancer Medicine, University of Liverpool, Liverpool, United Kingdom; Department of Nutritional Sciences, University of Surrey, Guildford, United Kingdom; Division of Digestive Diseases, Imperial College London, London, United Kingdom; Division of Digestive Diseases, Imperial College London, London, United Kingdom; Department of Neuroscience, Surgery and Trauma, Blizard Institute, Queen Mary University of London, London, United Kingdom; Department of Nutritional Sciences, King's College London, London, United Kingdom; Department of Nutritional Sciences, King's College London, London, United Kingdom

**Keywords:** almonds, gut microbiota, bifidobacteria, gut transit time, SCFA, butyrate, mastication, particle size

## Abstract

**Background:**

Almonds contain lipid, fiber, and polyphenols and possess physicochemical properties that affect nutrient bioaccessibility, which are hypothesized to affect gut physiology and microbiota.

**Objectives:**

To investigate the impact of whole almonds and ground almonds (almond flour) on fecal bifidobacteria (primary outcome), gut microbiota composition, and gut transit time.

**Methods:**

Healthy adults (*n* = 87) participated in a parallel, 3-arm randomized controlled trial. Participants received whole almonds (56 g/d), ground almonds (56 g/d), or an isocaloric control in place of habitual snacks for 4 wk. Gut microbiota composition and diversity (16S rRNA gene sequencing), SCFAs (GC), volatile organic compounds (GC-MS), gut transit time (wireless motility capsule), stool output and gut symptoms (7-d diary) were measured at baseline and endpoint. The impact of almond form on particle size distribution (PSD) and predicted lipid release was measured (*n* = 31).

**Results:**

Modified intention-to-treat analysis was performed on 79 participants. There were no significant differences in mean ± SD abundance of fecal bifidobacteria after consumption of whole almonds (8.7% ± 7.7%), ground almonds (7.8% ± 6.9%), or control (13.0% ± 10.2%; *q* = 0.613). Consumption of almonds (whole and ground pooled) resulted in higher mean ± SD butyrate (24.1 ± 15.0 μmol/g) than control (18.2 ± 9.1 μmol/g; *P* = 0.046). There was no effect of almonds on gut microbiota at the phylum level or diversity, gut transit time, stool consistency, or gut symptoms. Almond form (whole compared with ground) had no effect on study outcomes. Ground almonds resulted in significantly smaller PSD and higher mean ± SD predicted lipid release (10.4% ± 1.8%) than whole almonds (9.3% ± 2.0%; *P* = 0.017).

**Conclusions:**

Almond consumption has limited impact on microbiota composition but increases butyrate in adults, suggesting positive alterations to microbiota functionality. Almonds can be incorporated into the diet to increase fiber consumption without gut symptoms.

This trial was registered at clinicaltrials.gov as NCT03581812.

## Introduction

Diet is an important modifiable factor exerting a profound effect on the composition of the gut microbiota (1). The majority of randomized controlled trials (RCTs) exploring diet–microbiota interactions have focused on individual nutrients, with strong evidence for the modulatory impact of fiber (2). Fiber is derived from plant foods whose diverse components (macronutrients, micronutrients, nonnutrient bioactives) may interact to synergistically affect the gut microbiota, and their physical food matrix may alter nutrient availability for both host and micro-organisms (3). Considering the diverse roles of gut microbiota in human health (4), the identification of whole foods that affect the gut microbiota is important to inform public health messages and clinical practice guidelines.

Early studies suggested a prebiotic effect of almonds on fecal bifidobacteria in vitro (5–7) and in vivo (8). Subsequently, a systematic review of RCTs of nuts reported almond-specific effects including increased bacterial α-diversity and relative abundance of several bacteria at the genus level (9). However, there is a lack of consensus between RCTs on the impact of almonds on fecal bifidobacteria, β-diversity, and stool output that is likely due to limitations in study design that affect the assessment of gut microbiota, such as the use of a crossover design, which is not ideal because the effects of diet on microbiome can persist in the short term after discontinuation of the intervention (8) and because crossover trials can compromise blinding or masking of food interventions (10). Other limitations include washout periods of insufficient duration (11, 12) and lack of power (11–14). In addition, few studies addressed the impact of almond consumption on gut function or clinical outcomes (12, 13).

The mechanisms responsible for a prebiotic effect of almonds remain unclear, but potentially include their high fiber and polyphenol content and their low lipid bioaccessibility, due to their storage of lipids as droplets within tough cell walls that are resistant to human digestion (15). After mastication of whole almonds, the incomplete fracture of cell walls (16, 17) results in almond cells and their intracellular lipid reaching the colon intact where they are available for metabolism by the microbiota. In contrast, it is hypothesized that processing into ground almonds (almond flour) would result in higher lipid bioaccessibility due to decreased particle size before consumption, and therefore less lipid delivery to the colon. Maintenance of an intact almond cell matrix may therefore represent a unique method of delivering a rich supply of fermentable nutrients to the gut microbiota.

To address the lack of consensus between studies regarding the prebiotic effect of almonds and the potential impact of processing on the effect, a parallel-design RCT was conducted, powered to investigate the impact of whole or ground almonds on abundance of fecal bifidobacteria (primary outcome), gut microbiota composition, and gut transit time. To elucidate components responsible for the previously observed effects, we further investigated the role of almond processing by comparing the effects of whole almonds (low bioaccessible lipid) and ground almonds (high bioaccessible lipid) on study outcomes.

## Methods

### Study population

Participants in the study (NCT03581812) were healthy adult males and females (aged 18–45 y), who consumed snacks regularly (self-reported ≥ 2/d) and did not follow a moderate- or high-fiber diet [<22 g/d; assessed by the Block Fruit/Vegetable/Fiber screener (18)]. Volunteers were excluded if they fulfilled any of the following criteria: allergy; intolerance; dislike or regular consumption of almonds as snacks; history of gastrointestinal disorders; history of another chronic medical condition; history of abuse of alcohol, drugs, or other medications; antibiotic therapy in the past 4 wk; ongoing therapy with medication known to affect gastrointestinal motility; presence of medical devices; pregnant; planning pregnancy or lactating; consumption of prebiotics or probiotic supplements in the past 4 wk; consumption of prebiotic or probiotic foods in the past 2 wk; very high physical activity levels [assessed by the international physical activity questionnaire (19)]; BMI (in kg/m^2^) <18.5 or >29.9; or unintentional weight loss in the past 6 mo.

Participants were recruited via circular emails to staff and students of King's College London and other London universities; advertisements in local newspapers, social media, and clinical trials databases; and recruitment posters and leaflets between June 2018 and September 2019. Written informed consent was obtained before enrollment in the study. Trial procedures were approved by the King's College London Research Ethics Committee (RESCM-17/18-5341) and conducted in accordance with the Declaration of Helsinki.

### Study design

This was a free-living, 4-wk, 3-arm, parallel-design RCT conducted as a snack-replacement study to minimize impact on habitual background food intake (**Supplemental Figure 1**). The study was powered to detect differences in the primary outcome of fecal bifidobacteria abundance between groups based on a previous observation of an effect of almonds in comparison with control in a nonrandomized human trial [10.47% compared with 9.75% (8)] with 90% power. A significance level (α) of 0.0166 was used to allow for a post hoc Bonferroni correction for multiple testing. This calculation resulted in the requirement of 72 participants (24/group), but with an anticipated attrition of 15%–20% we aimed to recruit 87 participants (29/group). The study was also powered to detect differences in whole-gut transit time (WGTT) based on a previous observation of the effect of fiber in comparison with control on WGTT [22.5 h compared with 31.4 h (20)] with 80% power and an attrition rate of 15%–20%. Thus, 48 participants (16/group) were required to undergo measurement of WGTT.

Volunteers were screened via telephone and in person. Eligible participants attended study visits at the beginning and end of the 4-wk intervention period for collection of data and samples, and, in a subgroup, measurement of WGTT. All study visits took place at the Metabolic Research Unit, King's College London.

At baseline, participants were randomly allocated to receive either whole almonds (56 g/d), ground almonds (56 g/d), or an energy-matched control snack muffin (2/d) using block randomization with a block size of 6. Randomization was conducted by an independent researcher using a randomization website (www.sealedenvelope.com) with sex (male, female), age (18–30, 31–45 y), and willingness to undergo measurement of WGTT (yes, no) as stratification variables. Allocations were concealed from study researchers in opaque sealed envelopes that were only opened after completion of baseline measurements.

Blinding of participants to the intervention was not feasible for numerous reasons: almonds are easily identifiable; it was impossible to design a placebo identical to almonds, but without any active components; and it was necessary to exclude participants who had an allergy/intolerance or dislike to almonds. However, all efforts were made to mask participants to the true intervention by advertising the trial as a “snack replacement trial” testing the impact of a variety of snack foods on gut health, and avoiding any mention of almonds in advertising materials. Researchers were blinded to intervention allocation for data analysis through recoding of participant data.

Supplemental Figure 1 shows the trial design and procedures. The primary outcome assessed at baseline and endpoint was relative abundance of fecal bifidobacteria, and secondary outcomes were fecal microbiota composition (phyla and genera) and diversity (α and β), fecal SCFAs, fecal volatile organic compounds (VOCs), WGTT, regional gut transit times, gut pH, stool output (frequency and consistency), and gut symptoms. Potential confounders were monitored at baseline and endpoint: diet, body composition, and BMI. Compliance and acceptability were assessed at the endpoint visit.

### Dietary interventions

Participants were required to consume study snacks in place of habitual snack foods, twice a day for 4 wk. Two almond arms were included: whole almonds (2 × 28 g/d) and ground almonds (2 × 28 g/d). This dosage was selected because 28 g is the established amount for a single serving of almonds and has been used in studies previously (14), and this duration was selected because it was considered sufficient to allow for changes to gut microbiota (21), while also allowing for adaptation to the increase in fiber from almonds, and accounting for the impact of hormone fluctuations associated with the menstrual cycle on metabolism and GTT (22, 23).

To facilitate consumption, participants were instructed to consume the ground almond intervention mixed with 15 mL of water. Whole and ground natural almonds with skins (Nonpareil) were provided (Almond Board of California). The control group received an isocaloric muffin intervention (2 × muffin/d) that was developed at King's College London and used as a control in previous studies (24). The muffins were designed to provide a macronutrient profile approximating the average nutrient intake from snacks (excluding fruit) from the UK National Diet and Nutrition Survey (25). Muffins were prepared by study researchers at King's College London. **Table 1** includes the macronutrient profiles for the snacks consumed in each arm.

**TABLE 1:** Nutritional composition of almonds (whole, ground) and control snack muffins^1^

	Whole almonds	Ground almonds	Control snack muffin^2^
Serving size	56 g	56 g	2 muffins
Energy, kcal	324	324	318
Protein, g	12	12	7
Fat, g	28	28	12
SFAs, g	2	2	5
MUFAs, g	17	17	5
PUFAs, g	5	5	2
Carbohydrate, g	12	12	44
Sugars, g	2	2	24
Starch, g	9	9	20
Fiber, g	7	7	<1

1 Data from The Almond Board of California.

2 Based on the macronutrient profile of the average UK snack: 9.4% protein, 35.2% fat, 55.5% carbohydrate (25).

All participants were instructed to consume study snack interventions in place of usual snacks between meals and with a minimum of 100 mL water (26). Participants were asked to avoid consuming nuts, prebiotics, probiotics, and additional snacks, and to maintain habitual diet, exercise, and smoking habits.

### Stool sample collection and processing

Stool samples were collected by participants according to the Standard Operating Procedure for Fecal Sample Self-collection (27), delivered to investigators, and processed within 4 h. Briefly, samples were kept on ice, homogenized for 2 min each side (Steward Laboratory Blender Stomacher 400), divided into aliquots for later analyses, and stored at −80°C.

### DNA extraction, sequencing, and taxonomic analysis

DNA was extracted from stool using the DNEasy PowerLyser PowerSoil DNA Isolation Kit (Qiagen). DNA quality and quantity were confirmed using Nanodrop™ (Thermo Fisher Scientific). Sample libraries were prepared by amplifying the V1–V2 region of the 16S rRNA gene following the 16S Metagenomic Sequencing Library Preparation protocol (28) with the following modification: the index PCR reactions were cleaned and normalized using the SequalPrep normalization plate kit (Life Technologies). Sample libraries were quantified using the NEBNext library quant kit for Illumina (New England Biolabs). Sequencing was performed on an Illumina MiSeq platform (Illumina) using the MiSeq reagent kit V3 (Illumina) with paired-end 300 bp chemistry. Raw sequencing data were processed following the DADA2 pipeline in R (29).

Taxonomy was assigned using the SILVA database version 132 (30). Bacterial sequences were rarefied to an even sampling depth of 3195 sequences/sample. To reduce noise in the data caused by the presence of low-abundance/rare strains, a filter was applied to remove amplicon sequence variants (ASVs) with abundance <0.1% and presence in <10% of all samples. Centered log ratio transformation was applied before statistical analysis.

Analyses of bacterial diversity were conducted on processed sequencing data both before and after the application of the filter, to test the effect of removal of low-abundance/rare strains on diversity indices. Because both analyses produced similar results, and to maintain consistency with taxonomic comparisons, results are presented for filtered data only. α-Diversity was measured using the Chao1 index, Shannon's index, and Simpson's index (31) and β-diversity was calculated using unweighted and weighted UniFrac and Bray–Curtis dissimilarity (14, 32).

### Short-chain fatty acids

SCFAs were extracted using a buffer [0.1% HgCl_2_ (Sigma), 1% H_3_PO_4_ (Merck), 4.5% of the internal standard 2,2-dimethylbutyric acid (Sigma)]. The extracted SCFAs were quantified by GLC performed on a 7890a Agilent technology GC system with flame ionization detector, and a BP21 25-m fused silica capillary column with a 220-μm internal diameter and a film thickness of 0.25 μm (Trajan Scientific and Medical). The carrier gas was N_2_, and the oven was initially set to 80°C and programmed to increase by 10°C/min up to 145°C and 100°C/min up to 200°C to complete the elution. The injected sample volume was 0.2 μL, followed by a 1.2% formic acid wash solution (Merck) to minimize carryover from the previous sample. A blend of 6 pure SCFAs at 6 different concentrations were first run to produce calibration curves for quantitative analysis. All samples were analyzed in duplicate. The concentrations of individual SCFAs were expressed as μmol/g of wet feces and total SCFA concentrations were calculated as the sum of the individual SCFA concentrations.

### Volatile organic compounds

VOCs were extracted from fecal samples by solid phase micro-extraction coupled to GC-MS as described in detail previously (33).

### Gut transit time and luminal pH

Whole and regional gut transit times and pH were measured using the SmartPill® wireless motility capsule (WMC; Medtronic). The procedure for administration and analysis of the WMC has been described previously (34). In brief, the WMC was ingested orally and continuously measured gastrointestinal pH, temperature, and pressure via sensors encapsulated in an indigestible polyurethane shell. Data from sensors were transmitted to a receiver worn by the participant. Two researchers independently identified the following landmarks using proprietary software (MotiliGI™, Medtronic): ingestion (abrupt drop in pH, rapid rise in temperature); pyloric–duodenal transition (sharp, sustained rise in pH of >3 pH units); passage through the ileo-cecal junction (ICJ) (fall in pH of ≥1 pH unit, sustained for ≥10 min); and pill exit (sharp drop in temperature with termination of pressure and pH signals). Whole and regional gut transit times were derived from time intervals between landmarks: gastric emptying time (GET; from ingestion to pyloric–duodenal transition), small bowel transit time (SBTT; from pyloric–duodenal transition to passage through the ICJ), colonic transit time (CTT; from passage through the ICJ to pill exit), and WGTT (from ingestion to pill exit). Mean values for regional transit times and pH in the small bowel and colon, calculated using data from both researchers, were used for analysis. Normal ranges for transit times and pH have been published previously (35).

### Stool output and symptoms

Participants completed two 7-d stool and symptom diaries, incorporating the Bristol Stool Form Scale (BSFS) for assessment of stool output (36) and the Gastrointestinal Symptoms Rating Scale (GSRS) for assessment of common gastrointestinal symptoms (37, 38). Summary measures for stool output were stool frequency (total bowel movements per week), stool consistency (mean BSFS score over 7 d), and normal stools (proportion of stool types 3, 4, or 5 over 7 d).

The GSRS consists of 16 items rated on a Likert scale in terms of severity (0, absent; 1, mild; 2, moderate; 3, severe) and was measured at the end of each day for 7 d. Incidence was calculated as the number of days of mild, moderate, or severe symptoms and severity was calculated as the mean score over 7 d.

### Monitoring of confounders, quality of life, and compliance

Participants completed two 7-d estimated food diaries, 1 at baseline immediately before the intervention and 1 during the final week of the intervention. Diaries were the standard food diaries used in the UK National Diet and Nutrition Survey, including detailed instructions and visual aids to assist completion (39). Data from food diaries were entered into nutrition analysis software (Nutritics research edition, version 5.6; Nutritics) for analysis based upon the McCance & Widdowson composition of foods integrated data set.

Body weight and body composition were measured using bioelectrical impedance (BC-410MA; Tanita Ltd.). Height was measured using a wall-mounted stadiometer for calculation of BMI.

Health-related quality of life (QoL) was measured at baseline and endpoint using the short-form (SF)-36 questionnaire (40).

Participants were contacted weekly by telephone to encourage compliance. At the final visit participants returned all unused snack foods. Adequate compliance was defined as the consumption of ≥75% of snacks (≥42 snacks) because this is a common compliance threshold and would provide >5 g fiber/d. Participants who fulfilled this criterion were included in both intention-to-treat (ITT) and per-protocol (PP) analyses. Those who consumed <75% of snacks (<42 snacks) were included in the ITT analyses only. At the final visit participants completed an acceptability questionnaire developed for use in dietary intervention studies at King's College London.

### Mastication study

Participants had to opt in to take part in the mastication phase of the study, which required an additional study visit. The objective of this study was to assess the impact of almond form (whole almonds compared with ground almonds) on particle size distribution (PSD) and lipid release after mastication as an exploratory outcome.

Mastication sample collection and measurement of PSDs by mechanical sieving were conducted as described previously (41). Almonds were consumed in the same form (whole almonds or ground almonds mixed in 15 mL water) as in the snack provided in the feeding study. For mechanical sieving, the following sieve aperture sizes were used: 3350, 2000, 1000, 500, 250, 125, 63, 45, 20 µm (Endecotts Ltd.), and the proportion of masticated almonds retained on each sieve was calculated (% weight). Lipid bioaccessibility was predicted using a theoretical model developed and validated previously (17, 41, 42).

### Statistical analysis

For the majority of study outcomes statistical analysis was performed using IBM SPSS Statistics version 26 (IBM). All data were checked for normality and outliers using Q-Q plots and the Shapiro–Wilk statistic. Descriptive statistics were calculated: mean ± SD or median [IQR] for continuous outcomes and *n* (%) for categoric variables.

Differences between the 3 groups at endpoint were assessed using an ANCOVA, with baseline values as a covariate, or an ANOVA for change from baseline values. Where the tests were significant, comparisons on 2 groups were performed using a Bonferroni post hoc correction. For nonnormally distributed data, the Kruskal–Wallis test with post hoc Mann–Whitney test was applied. Categoric variables were assessed using the chi-square test. The following planned contrasts were conducted: *1*) analysis of almond groups pooled (whole almond and ground almond) compared with control; and *2*) analysis of whole almonds compared with ground almonds. Groups were compared by independent-samples *t* test or Mann–Whitney test.

The primary analysis was based on the ITT data set consisting of all participants randomly assigned. A modified-ITT analysis set (laboratory ITT), consisting of participants who provided sufficient stool at baseline and endpoint such that laboratory analyses could be completed, was used for the following outcomes: gut microbiota composition and diversity, SCFAs, and VOCs. A PP data set consisted of participants who completed the trial, maintained adequate compliance (>75%), and provided primary outcome data (stool). Missing data were assumed to be completely missing at random and no imputation was performed.

Analyses of gut microbiota composition were conducted using the MicrobiomeAnalyst software package (43). Taxonomic comparisons were conducted on relative abundance of taxa at the phylum and genus levels with differences between groups assessed by nonparametric tests. *P* values were corrected for multiple comparisons using the Benjamini–Hochberg false discovery rate (FDR) (*q*). A UniFrac weighted distance matrix was generated using the phangorn package in R (44) that was used to create nonmetric multidimensional scaling plots and permutational multivariate ANOVA *P* values using the Vegan library package in R (45).

Statistical analysis of fecal VOCs was conducted in Metaboanalyst version 4.0 (46). Missing data were replaced by one-fifth the minimum value for each compound. The data were normalized to the sample median, log transformed, and then auto-scaled. Differences in abundance of fecal VOCs between groups at baseline and endpoint were analyzed by 1-factor ANOVA and corrected for multiple testing using the Benjamini–Hochberg FDR (*q*). Principal component analysis (PCA) plots generated from log-transformed data were used to visually compare VOC profiles between groups.

Differences in PSDs from mechanical sieving were assessed by 2-factor repeated-measures ANOVA with sieve aperture size and almond form (whole or ground) as factors. Where there was a significant interaction, simple main effects were analyzed at each level of particle size (paired *t* test) and *P* values were corrected for multiple comparisons using a Bonferroni post hoc correction. Paired *t* test was used to detect differences in predicted lipid bioaccessibility between whole and ground almonds.

For all analyses *P* values and *q* values of <0.05 were considered statistically significant.

## Results

### Recruitment and participant characteristics

**FIGURE 1:**
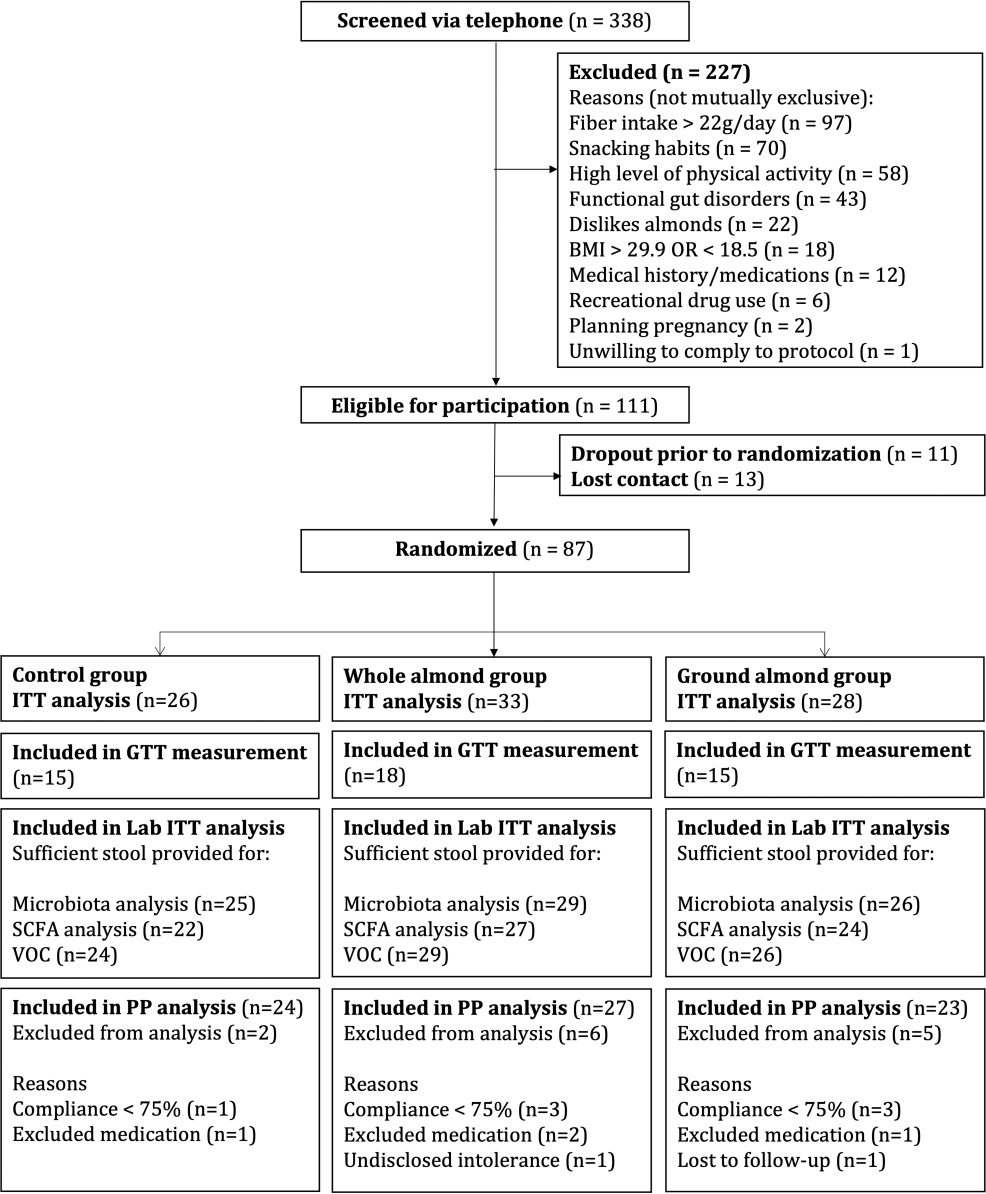
CONSORT diagram. GTT, gut transit time; ITT, intention-to-treat; PP, per-protocol; VOC, volatile organic compound.

Three hundred and thirty-eight volunteers were screened for eligibility; 87 were randomly assigned and included in the ITT analysis, of which 81 completed the intervention (CONSORT diagram in **Figure 1**). The numbers of participants included in the modified-ITT group for laboratory analysis were as follows: fecal microbiota, *n* = 80; SCFAs, *n* = 73; and VOCs, *n* = 79. A subgroup of 48 participants undertook measurement of gut transit time, pH, and pressure using the WMC, with 47 participants completing the test at baseline and 41 participants completing at endpoint. A subgroup of 31 participants opted to take part in the mastication analysis.

**Table 2** presents the demographic characteristics and baseline dietary intakes of the total cohort. The majority of eligible participants were female (86.2%), with a mean ± SD age of 27.5 ± 6.2 y, BMI of 22.9 ± 2.8, and fiber intake of 20.7 ± 7.7 g/d (from baseline food diary). There were no differences between study groups in baseline characteristics or baseline outcome measurements (with the exception of baseline relative abundance of the genus *Oscillibacter*).

**TABLE 2:** Demographic characteristics of participants^1^

	All (*n* = 87)	Control (*n* = 26)	Whole almond (*n* = 33)	Ground almond (*n* = 28)
Age, y	27.5 ± 6.2	27.9 ± 5.0	27.5 ± 6.8	27.0 ± 6.5
Female, *n* (%)	75 (86.2)	25 (96.2)	27 (81.8)	23 (82.1)
Weight, kg	63.9 ± 10.1	65.0 ± 8.1	64.4 ± 11.9	62.4 ± 9.6
BMI, kg/m^2^	22.9 ± 2.8	23.6 ± 2.7	22.6 ± 2.9	22.7 ± 2.9
Fat, %	27.9 ± 7.1	29.9 ± 6.0	26.9 ± 6.7	27.2 ± 8.2
FFM, kg	45.3 ± 5.5	45.3 ± 4.5	45.6 ± 6.2	45.0 ± 5.8
Energy, kJ	8143.1 ± 1949.8	8148.8 ± 2006.8	8205.1 ± 2021.7	8064.7 ± 1877.4
Energy, kcal/d	1941.3 ± 465.4	1942.3 ± 479.2	1956.7 ± 482.4	1922.2 ± 448.0
Protein, g/d	75.4 ± 20.3	74.6 ± 21.1	72.7 ± 19.6	79.3 ± 20.4
Fat, g/d	81.0 ± 23.8	79.1 ± 24.8	84.9 ± 26.1	78.2 ± 20.0
Carbohydrate, g/d	214.9 ± 64.0	218.7 ± 63.8	214.6 ± 66.6	211.7 ± 63.1
Fiber intake, g/d	20.7 ± 7.7	20.9 ± 7.8	21.9 ± 8.4	19.2 ± 6.7

1 Values are mean ± SD unless otherwise indicated.

### Compliance

Seven (8.0%) participants were noncompliant (consumption of <75% of snacks) to the whole almond (*n* = 3), ground almond (*n* = 3), or control muffin (*n* = 1) regime. Therefore, 74 participants were included in the PP analysis set. There were no differences in the number of compliant participants between groups (*P* = 0.464; chi-square test).

Overall compliance was 86.7% ± 27.7% corresponding to mean consumption of 49 ± 15.5 snacks throughout the intervention period. Compliance in the control group (93.5% ± 21.8%) was significantly greater than in the ground almond group (80.7% ± 27.8%; *P* = 0.028).

### Fecal microbiota composition

A total of 2,697,014 high-quality paired 16S rRNA gene sequences were obtained from all samples, an average of 16,149/stool sample (range: 3195–41,293). Sequences were rarefied to an even sampling depth of 3195 sequences/sample and resolved into a total of 9131 ASVs. After the application of filters to remove low-abundance and rare taxa, 417 ASVs remained and were included in analysis. The modified-ITT analysis consisted of 79 participants at the end of the intervention, owing to removal of 1 participant (ground almond group) for insufficient sequencing quality.

There were no significant differences in phyla or genera between groups at baseline, except for the genus *Oscillibacter* (*q* = 0.042), which was significantly higher in the control group (0.115% ± 0.132%) than in the whole almond group (0.024% ± 0.087%; *P* = 0.005).

In contrast to the primary hypothesis that almonds would increase the abundance of fecal bifidobacteria, there was significantly lower abundance after whole almonds (8.7% ± 7.7%) and ground almonds (7.8% ± 6.9%) than after control muffins (13.0% ± 10.2%; *P* = 0.031, Kruskal–Wallis test). However, this did not remain significant after FDR adjustment (*q* = 0.613; **Supplemental Table 1**). An additional 4 taxa were significantly different across the groups (*Lachnospiraceae_UCG_001, Phascolarctobacterium, Tuzzerella, Tyzzerella*; all *P* < 0.05). However, there were no significant differences for any bacteria at the phylum or genus level after FDR adjustment (all *q* > 0.05; Supplemental Table 1). There were no significant differences between groups for any taxa under the PP analysis or the planned contrasts.

**FIGURE 2:**
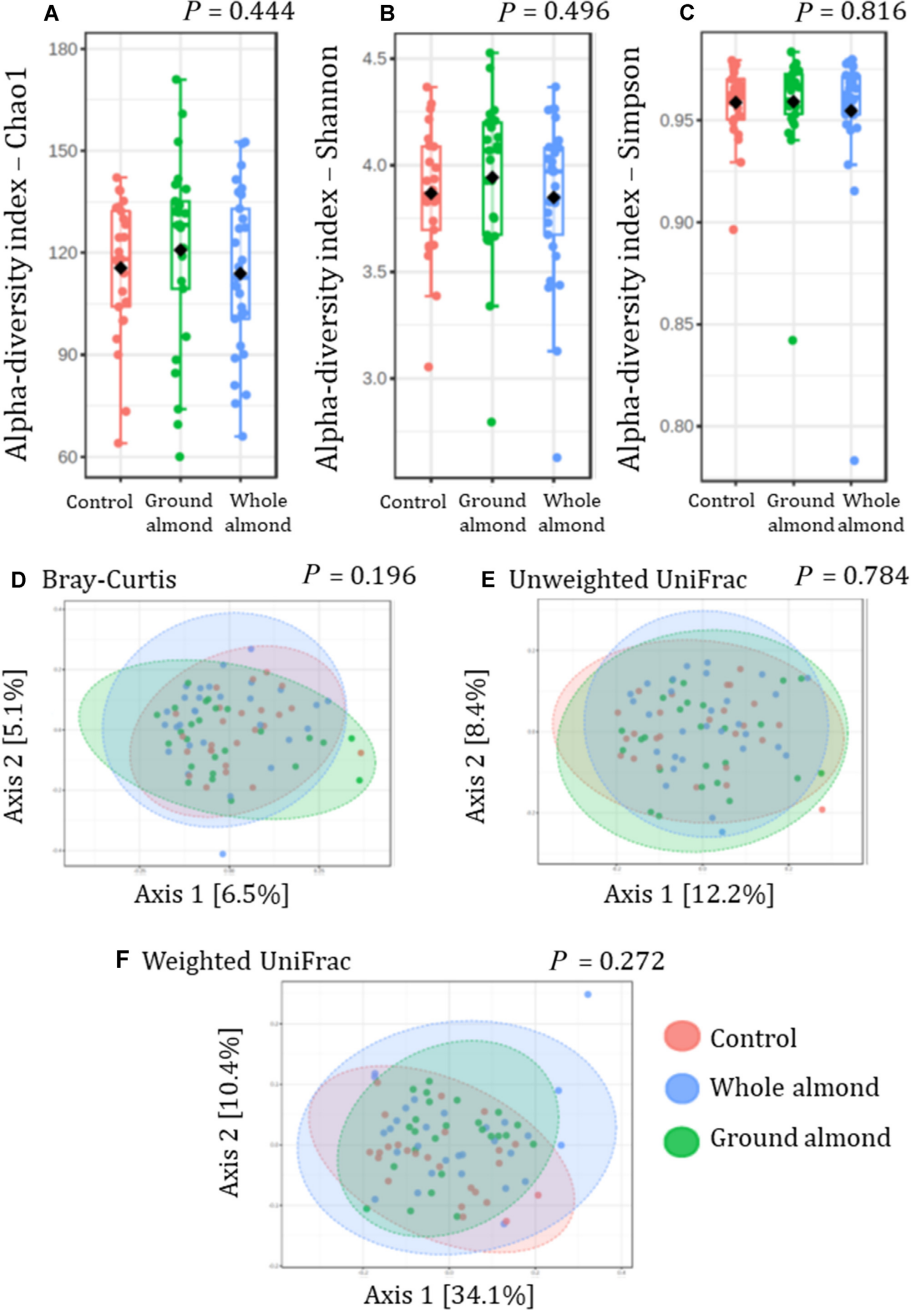
Gut microbiota diversity indices at the end of the intervention. All indices were calculated on rarefied abundance data after application of a filter to remove low-abundance/rare taxa. (A–C) α-Diversity indices: (A) Chao1 index; (B) Shannon's index; (C) Simpson's index. Boxplots are median [IQR] with the sample mean represented by the black diamond. Individual sample values are represented by colored dots. *P* values are the results of Kruskal–Wallis tests. (D–F) β-Diversity indices: (D) Bray–Curtis dissimilarity; (E) unweighted UniFrac distance; (F) weighted UniFrac distance in the whole almond, ground almond, and control groups. *P* values are the result of permutational multivariate ANOVAs. Modified intention-to-treat, *n* = 79: control, *n* = 25; ground almond, *n* = 25; whole almond, *n* = 29.

There were no other significant differences observed in microbiota analyses, or α-diversity or β-diversity (**Figure 2**).

### Gut microbiota metabolites

In the ITT analysis, there were no significant differences between groups for total or individual SCFAs, when analyzed as either absolute or change in concentrations (μmol/g feces) (**Table 3**). However, in the PP analysis set, butyrate was significantly higher after almonds (whole and ground pooled; not included in table; 24.1 ± 15.0 μmol/g) than after control (18.2 ± 9.1 μmol/g; *P* = 0.046). For remaining sensitivity analyses, there were no significant differences between groups for any other SCFA.

**TABLE 3:** SCFAs (μmol/g wet feces) at baseline and end of intervention, and change from baseline to end of intervention in the modified-ITT and PP analysis^1^

	Control			Whole almond			Ground almond			*P* values	
	Baseline	Endpoint	Change	Baseline	Endpoint	Change	Baseline	Endpoint	Change	ANCOVA^2^	ANOVA^3^
ITT	*n* = 22	*n* = 22	*n* = 22	*n* = 27	*n* = 27	*n* = 27	*n* = 24	*n* = 24	*n* = 24		
Total SCFA	110.1 ± 40.8	122.1 ± 44.9	12.1 ± 45.8	142.2 ± 62.0	144.9 ± 65.2	2.7 ± 58.4	117.3 ± 52.5	127.1 ± 63.2	9.8 ± 66.1	0.915	0.836
Acetate	67.2 ± 25.9	72.5 ± 28.5	5.3 ± 30.3	82.1 ± 32.9	82.5 ± 32.8	0.5 ± 33.3	66.9 ± 29.2	71.9 ± 36.1	5.0 ± 40.7	0.858	0.859
Propionate	17.6 ± 6.7	21.1 ± 9.8	3.6 ± 7.9	23.5 ± 13.5	24.8 ± 12.3	1.3 ± 9.8	22.3 ± 11.9	22.5 ± 12.3	0.2 ± 13.5	0.826	0.562
Butyrate	15.5 ± 7.8	17.7 ± 9.0	2.2 ± 8.9	24.9 ± 16.0	25.5 ± 17.1	0.6 ± 14.7	16.8 ± 11.0	20.9 ± 12.8	4.2 ± 12.2	0.752	0.589
Isobutyrate	2.2 ± 1.5	2.3 ± 1.0	0.1 ± 1.2	2.6 ± 1.4	2.7 ± 1.7	0.1 ± 1.7	2.6 ± 1.4	2.5 ± 1.3	−0.0 ± 1.6	0.861	0.947
Valerate	3.0 ± 1.8	3.6 ± 1.6	0.6 ± 1.7	3.8 ± 2.5	3.9 ± 2.3	0.0 ± 2.1	3.4 ± 2.0	3.9 ± 2.3	0.6 ± 1.7	0.747	0.461
Isovalerate	4.6 ± 3.4	4.8 ± 2.5	0.2 ± 2.8	5.6 ± 3.2	5.6 ± 3.9	0.3 ± 3.4	5.4 ± 3.2	5.4 ± 2.9	−0.0 ± 3.5	0.847	0.945
PP	*n* = 22	*n* = 22	*n* = 22	*n* = 27	*n* = 25	*n* = 25	*n* = 21	*n* = 23	*n* = 21		
Total SCFA	109.5 ± 50.2	122.6 ± 44.7	11.0 ± 46.6	137.9 ± 62.9	148.5 ± 66.1	7.5 ± 58.0	117.5 ± 50.2	134.6 ± 63.6	10.2 ± 69.8	0.746	0.978
Acetate	66.7 ± 26.5	72.2 ± 28.7	4.6 ± 30.9	78.8 ± 33.3	84.3 ± 33.2	3.6 ± 32.5	68.3 ± 29.3	77.6 ± 39.3	4.5 ± 42.7	0.717	0.994
Propionate	17.5 ± 6.7	21.1 ± 9.8	3.5 ± 8.1	22.8 ± 13.7	25.3 ± 12.6	2.0 ± 9.8	21.7 ± 11.2	23.7 ± 12.8	0.6 ± 13.6	0.760	0.683
Butyrate	15.5 ± 7.7	18.2 ± 9.1	2.0 ± 9.0	24.1 ± 16.3	26.3 ± 17.4	1.5 ± 14.9	16.2 ± 9.2	21.7 ± 11.7	4.5 ± 13.0	0.631	0.694
Isobutyrate	2.2 ± 1.5	2.4 ± 1.1	0.1 ± 1.2	2.7 ± 1.4	2.8 ± 1.8	0.1 ± 1.7	2.5 ± 1.3	2.5 ± 1.0	−0.0 ± 1.7	0.717	0.921
Valerate	3.0 ± 1.8	3.6 ± 1.6	0.6 ± 1.7	3.9 ± 2.4	4.0 ± 2.3	0.1 ± 2.2	3.4 ± 1.7	4.0 ± 2.1	0.7 ± 1.8	0.752	0.506
Isovalerate	4.6 ± 3.4	5.1 ± 2.7	0.2 ± 2.9	5.5 ± 3.3	5.8 ± 4.0	0.3 ± 4.0	5.3 ± 2.8	5.2 ± 2.3	−0.1 ± 3.7	0.740	0.926

1 All values are mean ± SD unless otherwise indicated. Numbers in each group: modified ITT, *n* = 73; PP, *n* = 74. ITT, intention-to-treat; *n*, number of participants with available data; PP, per-protocol.

2 ANCOVA is the *P* value after comparison of endpoint values with baseline values as a covariate.

3 ANOVA is the *P* value after comparison of change values; ITT population was modified to include only those who provided sufficient sample for analysis.

**FIGURE 3:**
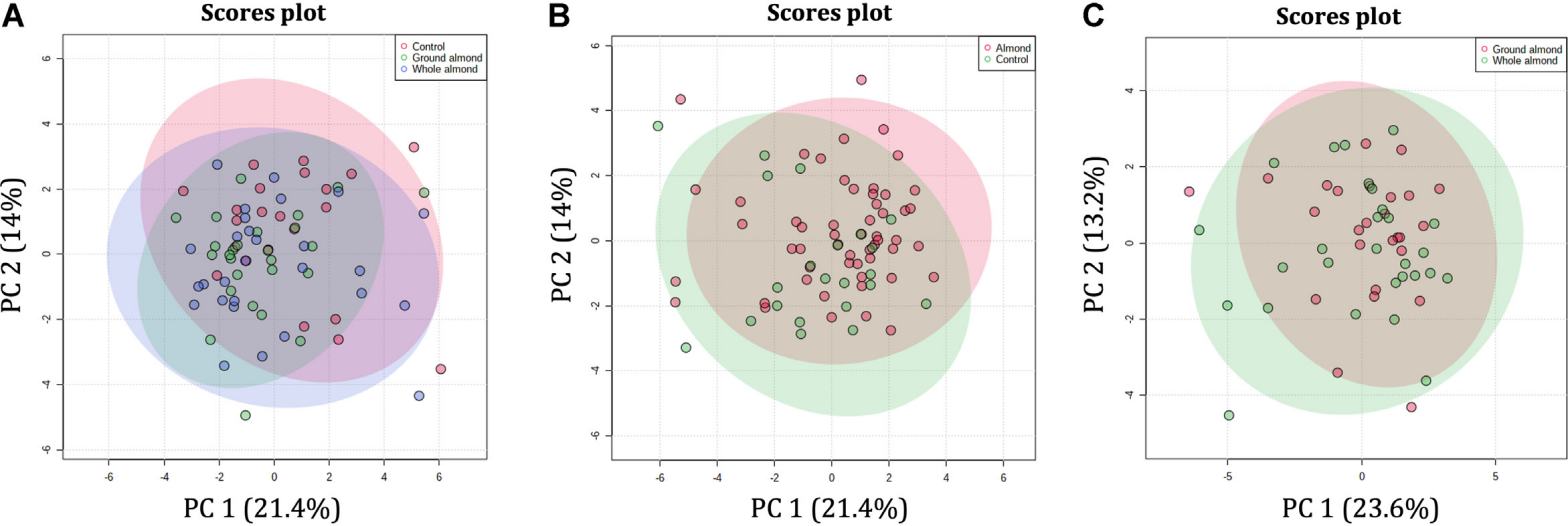
PC analysis plot of fecal volatile organic compound profile in groups that consumed whole almond, ground almond, or control muffin (A); almond (whole and ground pooled) or control (B); and whole almond or ground almond (C). Modified intention-to-treat, *n* = 76: control, *n* = 22; ground almond, *n* = 26; whole almond, *n* = 28. PC, principal component.

After removal of low-copy features from raw data, 118 VOCs were identified from all samples. PCA plots at baseline and endpoint (**Figure 3**) revealed no evidence of clustering of groups based on VOC profile. There were no differences between groups for any individual VOCs identified at baseline or endpoint.

Planned contrasts were conducted to compare almonds (whole almond and ground almond groups pooled) with control muffins and there was no evidence of clustering of groups from the PCA plot (Figure 3). Three VOCs were increased after almond consumption (whole and ground pooled) in comparison with control muffins: 2-methylbutanoic acid (*P* = 0.004, *q* = 0.057), 3-methylbutanoic acid (*P* = 0.006, *q* = 0.057), and nonanal (*P* = 0.007, *q* = 0.057), which even after correction for multiple testing (FDR) approached traditional statistical significance (**Supplemental Table 2**). Comparison of the whole almond and ground almond groups revealed no evidence of clustering of groups from the PCA plots (Figure 3) and no difference between groups for any VOCs after correction for multiple testing.

### Gastrointestinal transit time and pH

Comparison between the 3 groups at the end of the intervention revealed no significant differences in WGTT (*P* = 0.940) (**Table 4**). Similarly, no significant differences between groups were identified for GET, SBTT, or CTT. There were no significant differences for any transit outcome variable in the PP analysis or planned contrasts (Table 4).

**TABLE 4:** Whole and regional gut transit times and gastrointestinal pH at baseline and endpoint in the ITT and PP populations^1^

	Control				Whole almond				Ground almond				*P* values		
	*n*	Baseline, hh:mm	*n*	Endpoint, hh:mm	*n*	Baseline, hh:mm	*n*	Endpoint, hh:mm	*n*	Baseline, hh:mm	*n*	Endpoint, hh:mm	ANCOVA	Pooled almond vs. control^2^	Whole vs. ground^2^
ITT															
WGTT	14	36:28 ± 20:43	13	35:06 ± 18:02	18	40:46 ± 25:56	15	34:31 ± 12:51	15	34:17 ± 16:25	13	36:12 ± 23:20	0.940	0.955	0.487
GET	14	02:46 ± 00:37	12	04:17 ± 04:32	18	02:38 ± 00:43	15	03:18 ± 01:35	15	02:52 ± 00:55	13	03:32 ± 04:14	0.769	0.082	0.442
SBTT	14	05:21 ± 01:18	12	05:34 ± 00:52	18	05:14 ± 01:27	14	05:01 ± 01:01	14	04:15 ± 01:09	13	04:34 ± 01:22	0.325	0.055	0.349
CTT	14	28:20 ± 19:55	12	26:19 ± 19:36	18	32:46 ± 25:20	15	25:52 ± 12:20	14	27:56 ± 16:37	13	28:05 ± 21:03	0.578	0.637	0.906
Small bowel pH	14	7.1 ± 0.3	12	7.2 ± 0.4	18	7.1 ± 0.3	14	7.1 ± 0.4	14	7.1 ± 0.3	13	7.3 ± 0.3	0.578	0.763	0.331
Colonic pH	13	6.7 ± 0.8	12	6.5 ± 0.7	18	6.5 ± 0.6	14	6.5 ± 0.5	13	6.6 ± 0.7	13	6.5 ± 0.8	0.937	0.819	0.845
PP															
WGTT	12	38:58 ± 21:26	12	35:18 ± 18:49	14	41:39 ± 26:58	14	35:22 ± 12:54	12	28:57 ± 11:17	12	33:14 ± 21:41	0.728	0.936	0.241
GET	12	02:42 ± 00:38	11	02:59 ± 00:28	14	02:43 ± 00:44	14	03:22 ± 01:37	12	02:46 ± 00:56	12	02:22 ± 00:40	0.091	0.076	0.181
SBTT	12	05:34 ± 01:16	11	05:31 ± 00:53	14	05:19 ± 01:30	13	05:02 ± 01:04	11	04:24 ± 01:02	12	04:31 ± 01:25	0.548	0.091	0.301
CTT	12	30:41 ± 20:40	11	27:59 ± 19:38	14	33:36 ± 26:31	14	26:36 ± 12:27	11	22:16 ± 11:13	12	26:21 ± 20:59	0.875	0.974	0.584
Small bowel pH	12	7.1 ± 0.3	11	7.1 ± 0.4	14	7.2 ± 0.3	13	7.1 ± 0.4	11	7.1 ± 0.4	12	7.3 ± 0.3	0.370	0.467	0.238
Colonic pH	11	6.8 ± 0.8	11	6.5 ± 0.7	14	6.5 ± 0.6	13	6.4 ± 0.6	10	6.6 ± 0.7	12	6.5 ± 0.8	0.878	0.746	0.851

1 All values are mean ± SD unless otherwise indicated. *P* values are the result of ANCOVA with baseline values as a covariate. CTT, colonic transit time; GET, gastric emptying time; ITT, intention-to-treat; *n*, number of participants with available data; PP, per-protocol; SBTT, small bowel transit time; WGTT, whole-gut transit time.

2 *P* values are the result of an independent-samples *t* test on endpoint values.

There were no significant differences in small bowel pH or colonic pH between the control muffins, whole almond, and ground almond groups, or in the planned contrasts in either the ITT or PP analyses (Table 4).

### Stool output and symptoms

**Table 5** presents stool frequency and stool consistency (BSFS score and % normal stools). There was a significant difference in change in stool frequency at the end of the intervention period (*P* = 0.017), with greater change in the whole almond group (+1.5; IQR: −1.5−4.5) than in the control group (−1.0; IQR: −4.0−2.0; *P* = 0.034) or ground almond group (−0.5; IQR: −4.8−3.8; *P* = 0.061). No other significant differences were observed. There were no group differences in incidence or severity of common gastrointestinal symptoms (**Supplemental Tables 3** and **4**).

**TABLE 5:** Stool frequency in the ITT population^1^

	Control			Whole almond			Ground almond			*P* values	
	Baseline (*n* = 26)	Endpoint (*n* = 25)	Change^2^ (*n* = 25)	Baseline (*n* = 33)	Endpoint (*n* = 30)	Change^2^ (*n* = 30)	Baseline (*n* = 28)	Endpoint (*n* = 26)	Change^2^ (*n* = 26)	ANCOVA^3^	Kruskal–Wallis^4^
Stool frequency, BMs/wk	8.3 ± 3.9	7.7 ± 3.1	−1.0 [−4.0−2.0]	7.9 ± 3.5	9.2 ± 3.7	1.5 [−1.5−4.5]^5^	9.4 ± 3.5	9.1 ± 4.6	−0.5 [−4.8−3.8]	0.070	0.017
BSFS score	4.0 ± 0.7	3.7 ± 0.8	−0.3 [−1.1−0.5]	3.9 ± 1.0	3.8 ± 0.8	0.0 [−1.5−1.5]	3.8 ± 0.6	3.8 ± 0.8	0.0 [−0.7−0.7]	0.508	0.368
Normal stools, %	88.2 ± 34.1	85.7 ± 36.1	0.0 [−25.3−25.3]	77.8 ± 47.2	89.2 ± 35.0	1.4 [−23.6−26.4]	87.1 ± 32.7	97.1 ± 25.9	0.0 [−22.4−22.4]	0.617	0.213

1 Values are mean ± SD unless otherwise indicated. Numbers in each group: ITT, *n* = 87: control, *n* = 26; whole almond, *n* = 33; ground almond, *n* = 28. BM, bowel movement; BSFS, Bristol Stool Form Scale; ITT, intention-to-treat; *n*, number of participants with available data.

2 Values are median [IQR].

3 *P* values are the result of ANCOVA on endpoint values with baseline values as a covariate.

4 *P* values are the result of a Kruskal–Wallis H test on change values.

5 Significantly greater change than control, *P* = 0.034.

### Nutrient intake

At the end of the intervention there were significant differences between the groups in intakes of many nutrients (**Supplemental Table 5**), with post hoc testing revealing intakes of absolute energy, protein, total fat, MUFA, total fiber, non-starch polysaccharides, potassium, magnesium, phosphorus, zinc, and manganese were higher in the whole almond group than in the control muffins group, whereas intakes of MUFA, total fiber, non-starch polysaccharides, magnesium, and manganese were higher in the ground almond group than in the control muffins group.

### Body composition

There were no significant differences between groups in body weight, BMI, or body composition at the end of the intervention (**Supplemental Table 6**) or for any domain assessed by the SF-36 questionnaire (**Supplemental Table 7**).

### Quality of life and acceptability

Most participants in both the whole almond and ground almond groups reported favorable opinions of the interventions’ flavor (75.9% and 41.7%, respectively) and portion size (63.3% and 38.5%, respectively). For whole almonds, the majority of participants had favorable opinions of the snacks’ texture (60%) and mouth feel (53.3%), in contrast to ground almonds, with many participants reporting a dislike of the snacks’ texture (42.3%) and mouth feel (36%).

### Mastication, particle size distribution, and lipid bioaccessibility

A total of 31 participants completed the mastication phase. Analysis of PSDs revealed a significant interaction between almond form (whole or ground) and particle size on PSD (*P* < 0.001).

**FIGURE 4:**
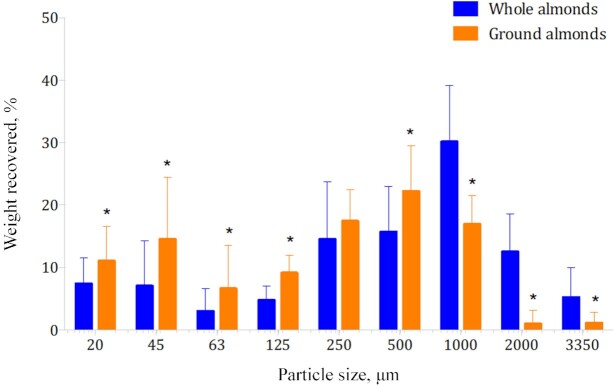
PSDs of masticated whole and ground almonds measured by mechanical sieving. Bars are mean weights recovered, error bars are SDs. *n* = 31 participants provided paired data. Significant interaction between almond form (whole or ground) and particle size on PSD from 2-factor repeated-measures ANOVA (*P* < 0.001). *Significant difference between whole and ground almonds after analyzing simple main effects (paired *t* test) at that level of particle size (*P* < 0.05). PSD, particle size distribution.

After mechanical sieving, significantly more smaller particles were retained on sieves for masticated ground almonds than for whole almonds (20 µm, *P* = 0.009; 45 µm, *P* = 0.018; 63 µm, *P* < 0.001; 125 µm, *P* < 0.001; 500 µm, *P* < 0.001) and significantly more larger particles were retained for whole almonds than for ground almonds (1000 µm, *P* < 0.001; 2000 µm, *P* < 0.001; 3350 µm, *P* < 0.001) (**Figure 4**).

Lipid bioaccessibility of masticated whole and ground almonds predicted using a theoretical model (42) revealed a significantly greater lipid bioaccessibility for masticated ground almonds (10.4% ± 1.8%) than for masticated whole almonds (9.3% ± 2.0%; *P* = 0.017).

## Discussion

In this first appropriately powered, parallel-design RCT to investigate the impact of almonds and almond processing on gut microbiota and incorporating clinical and gut function endpoints, we observed that almonds may affect gut microbial metabolism and stool output. However, contrary to our hypothesis, results of this RCT show that consumption of whole or ground almonds for 4 wk had no impact on fecal bifidobacteria numbers; indeed, the abundance was numerically lower in the almond groups. These findings are in agreement with previous RCTs that have reported no effect of almond consumption on bifidobacteria (13, 14), but are in contrast to 1 RCT that found a significant decrease in bifidobacteria after a pooled analysis of 4 processed almond forms (whole natural, whole roasted, chopped, butter) in comparison with control (11). As outlined, previous RCTs had significant limitations, which were overcome in the current trial and therefore our results can be considered robust.

There were no significant differences between groups for any taxa at the phylum or genus level at the end of the intervention period. This is despite good compliance to whole almonds (86.5%, ∼48 g/d) and ground almonds (80.7%, ∼45 g/d). It is widely acknowledged that gut microbiota composition is subject to large interindividual variability (47), therefore, although the RCT was powered to detect changes in bifidobacteria it is possible that secondary outcomes such as bacterial abundance at the phylum and genus levels were insufficiently powered to detect significant effects should they occur.

Several members of the family Lachnospiraceae were altered by almond consumption in this RCT, but the observed effects did not remain significant after correction for multiple testing. Lachnospiraceae are among the main producers of colonic SCFAs (48), and members of this family have been reported to be influenced by almond consumption in a meta-analysis of almond interventions (9). Interestingly, we also observed significant increases in the SCFA butyrate and several VOCs after almond consumption (whole and ground pooled) in comparison with control muffins in the first RCT to assess the impact of almonds on bacterial metabolites. It was anticipated that almonds would increase production of SCFA, which would result in a more acidic pH, particularly in the right side of the colon. However, contrary to these findings, there was no impact of almonds on colonic pH. Therefore, we must interpret these results with caution, owing to uneven group sizes and potential for type 1 error. Nonetheless, they indicate potentially important outcomes for future investigations. In particular, butyrate plays a role in multiple processes relating to human health (49). For example, 2-methylbutyric acid is produced by bacteria when carbohydrates are limited (50), indicating a transfer from saccharolytic to proteolytic metabolism potentially due to increased availability of almond proteins. Also, nonanal has been identified in roasted almonds after storage, and is considered an indicator of shelf life (51), representing an area for future investigation as an objective biomarker of almond intake.

There was no impact of almond consumption on α-diversity or β-diversity by any metric, in agreement with previous RCTs that also reported no effect of almonds on β-diversity (11, 14). In contrast, a previous RCT reported an effect of almonds on α-diversity (14), whereby snacking on almonds for 8 wk resulted in increases in both the Chao1 index and Shannon's index in comparison with control. Similarly, the meta-analysis of almond RCTs reported consumption of almonds resulted in an increase in Shannon's index that was borderline statistically significant (9). Despite these conflicting results, it is worth emphasizing that evidence for an effect of gut microbiota diversity on human health is limited to observational trials and therefore its importance as an outcome in dietary intervention trials remains unclear (52).

Duration of interventions is an important consideration in diet–microbiome studies, a factor that may explain the variability in results between studies (9). The long-term impact of almonds on gut microbiota remains to be evaluated. It has been suggested that although short-term changes in diet (2 d–12 wk) rapidly alter gut microbiota, it is possible that long-term changes in dietary habits (>6 mo) have the greatest potential influence on gut microbiota composition and subsequent clinical benefits associated with their modulation (53). Dose may be important as shown from a previous meta-analysis (9) but it was not possible to explore this in the current study.

This was, to our knowledge, the first RCT to investigate the impact of almonds on whole and regional gut transit time and luminal pH. Contrary to our hypothesis that almonds would result in faster WGTT, our findings indicate no effect on this outcome. A plausible explanation for this has been illustrated by a systematic review of 65 intervention trials that reported that the effect of fiber on transit is dependent on baseline WGTT, whereby reductions are most pronounced in those with baseline WGTT of >48 h (54). In this study, mean ± SD WGTT at baseline was normal [37.4 ± 21.4 h (35)], potentially accounting for the lack of overall effect of almonds on WGTT. Our results indicate that almonds have a small impact on increasing stool frequency in healthy people but have no effect on stool consistency (when assessed by both subjective and objective measures) or gut symptoms. This finding confirms that whole almonds and ground almonds, consumed as a snack twice a day for 4 wk, are well tolerated by healthy adults with low fiber intake.

Finally, almond processing had no impact on any study outcome. The hypothesis that, after mastication, ground almonds would have a PSD with greater proportions of smaller particles than would whole almonds, and this would subsequently influence predicted lipid bioaccessibility, was investigated in the mastication study. Our results confirmed that after mastication, ground almonds had more particles of smaller size than did whole almonds. Despite this, although it was significant, the difference in lipid bioaccessibility between these almond forms was very small (mean ± SD difference: 1.1% ± 2.3%). Therefore, these findings support the comparable effect of whole and ground almonds on study outcome measures. It can be concluded that commercial grinding of almonds does not result in clinically meaningful differences in nutrient bioaccessibility.

The main limitation of this trial is the sex distribution, which is predominantly female (86.2%) and young (27.5 ± 6.2 y), and therefore the results are not representative of male and older populations.

In conclusion, almond consumption does not exert a prebiotic effect on fecal bifidobacteria abundance or result in major changes in other gastrointestinal microbiota, gastrointestinal transit, pH, pressure, stool output, or gut symptoms in healthy adults. Therefore, their incorporation into the diet of low fiber consumers among the public, to increase fiber intake, would likely be well tolerated. Almond consumption may influence the family Lachnospiraceae and aspects of bacterial metabolism, in particular fecal butyrate. These outcomes warrant further investigation in future RCTs, which should focus on confirming these findings in cohorts of older adults with an even sex distribution. Commercial processing of almonds increases predicted lipid bioaccessibility to a limited degree but did not appreciably influence gut health.

## Supplementary Material

nqac265_Supplemental_Files

## Data Availability

Data described in the article, code book, and analytic code will be made available upon request pending application.

## Notes

Supported by an Almond Board of California grant (to KW). The funders provided financial support, and the whole and ground almonds consumed by participants in the trial, but had no role in study design, conduct, analysis, interpretation, or decision to publish. The Division of Digestive Disease at Imperial College London receives financial support from the National Institute of Health Research (NIHR) Imperial Biomedical Research Centre (BRC) based at Imperial College Healthcare National Health Service Trust and Imperial College London.

Author disclosures: ACC was funded by a PhD studentship funded by Almond Board of California. ED has received an education grant from Alpro, research funding from the British Dietetic Association, Almond Board of California, the International Nut and Dried Fruit Council, and Nestec Ltd, and has served as a consultant for Puratos. MR and KW have received research funding from Almond Board of California, Danone, and International Dried Fruit and Nut Council, and are co-inventors of volatile organic compounds in the diagnosis and dietary management of irritable bowel syndrome. MR is also cofounder of Bio&Me, a gut health food brand. TG supervises PhD students partially funded by Mondelez and McCain Foods Ltd, and has previously received research funding from Almond Board of California. SEB has received grant funding from Almond Board of California, Malaysian Palm Oil Board, and ZOE Ltd, and receives consultancy and options from ZOE Ltd. All other authors report no conflicts of interest.

Supplemental Tables 1–7 and Supplemental Figure 1 are available from the “Supplementary data” link in the online posting of the article and from the same link in the online table of contents at https://academic.oup.com/ajcn/.

Abbreviations used: ASV, amplicon sequence variant; BSFS, Bristol Stool Form Scale; CTT, colonic transit time; FDR, false discovery rate; GET, gastric emptying time; GSRS, Gastrointestinal Symptoms Rating Scale; ICJ, ileo-cecal junction; ITT, intention-to-treat; PCA, principal component analysis; PP, per-protocol; PSD, particle size distribution; QoL, quality of life; RCT, randomized controlled trial; SBTT, small bowel transit time; SF, short-form; VOC, volatile organic compound; WGTT, whole-gut transit time; WMC, wireless motility capsule.
