# Supplementary material for: The impact of almonds and almond processing on gastrointestinal physiology, luminal microbiology, and gastrointestinal symptoms: a randomized controlled trial and mastication study
Source: Am J Clin Nutr. 2022 Sep 20;116(6):1790–804. doi: 10.1093/ajcn/nqac265 (PMC9761756; doi:10.1093/ajcn/nqac265)
Supplement: nqac265_Supplemental_Files [file nqac265_supplemental_files.zip › Online supp for production post accceptance.docx]

**The impact of almonds and almond processing on gastrointestinal physiology, luminal microbiology and gastrointestinal symptoms: a randomized controlled trial and mastication study**

Alice C Creedon (1), Eirini Dimidi (1), Estella S Hung (1), Megan Rossi (1), Christopher Probert (2), Terri Grassby (3), Jesus Miguens-Blanco (4), Julian R Marchesi (4), S Mark Scott (5), Sarah E Berry (1), Kevin Whelan (1)

Online Supplementary Material

**Table of contents**

**TABLE 1 Fecal microbiota composition (% relative abundance) at end of the intervention in the modified intention to treat analysis set_________________ 3**

**TABLE 2 Table of volatile organic compounds significantly increased by almond consumption (pooled whole and ground) in comparison to control in the modified intention to treat population 8**

**TABLE 3 Incidence of gastrointestinal symptoms in the intention to treat population 9**

**TABLE 4 Severity of gastrointestinal symptoms in the intention to treat population 11**

**TABLE 5 Energy and nutrient intakes at baseline and endpoint in the intention to treat population 13**

**TABLE 6 Body weight and body composition at baseline and endpoint in the intention to treat population 17**

**TABLE 7 Quality of life in the intention to treat population 18**

**FIGURE 1 Summary of the study design, interventions and procedures of a randomized controlled trial investigating the impact of almonds (whole or ground) on gut microbiology, physiology and function in 87 healthy adults 19**

**SUPPLEMENTARY TABLE 1 Fecal microbiota composition (% relative abundance) at end of the intervention in the modified intention to treat analysis set**

|  | **Control muffin** | **Whole almond** | **Ground almond** | **Three group^1^** | | | **Pooled almond vs. control^2^** | | | **Whole vs. ground^2^** | | | |
| --- | --- | --- | --- | --- | --- | --- | --- | --- | --- | --- | --- | --- | --- |
|  | n = 25 | n = 29 | n = 25 | *p-*value | | *q-*value | *p-*value | | *q-*value | *p-*value | | *q-*value | |
| **Phyla** |  |  |  |  |  | |  |  | |  |  | |  |
| p_Firmicutes | 55.1 (14.0) | 60.7 (16.1) | 60.4 (12.5) | 0.279 | 0.520 | | 0.115 | 0.345 | | 0.744 | 0.787 | |  |
| p_Bacteroidetes | 25.7 (13.3) | 25.5 (14.6) | 26.2 (11.5) | 0.563 | 0.607 | | 0.464 | 0.464 | | 0.491 | 0.787 | |  |
| p_Actinobacteria | 16.7 (10.5) | 11.4 (8.5) | 11.0 (7.7) | 0.051 | 0.307 | | **0.018** | 0.111 | | 0.547 | 0.787 | |  |
| p_Proteobacteria | 0.6 (1.2) | 0.4 (0.6) | 0.3 (0.4) | 0.607 | 0.607 | | 0.328 | 0.464 | | 0.787 | 0.787 | |  |
| p_Verrucomicrobia | 0.1 (0.2) | 0.2 (0.7) | 0.1 (0.3) | 0.185 | 0.520 | | 0.348 | 0.464 | | 0.098 | 0.588 | |  |
| p_Desulfobacterota | 0.0 (0.1) | 0.0 (0.1) | 0.1 (0.1) | 0.347 | 0.520 | | 0.423 | 0.464 | | 0.253 | 0.759 | |  |
| **Genera** |  |  |  |  |  | |  |  | |  |  | |  |
| g_Actinomyces | 0.0 (0.0) | 0.0 (0.0) | 0.0 (0.1) | 0.239 | 0.797 | | 0.526 | 0.860 | | 0.928 | 0.941 | |  |
| g_Adlercreutzia | 0.1 (0.1) | 0.1 (0.3) | 0.3 (0.4) | 0.811 | 0.939 | | 0.207 | 0.783 | | 0.265 | 0.838 | |  |
| g_Agathobacter | 3.1 (4.7) | 4.2 (4.9) | 3.0 (3.1) | 0.848 | 0.939 | | 0.724 | 0.902 | | 0.690 | 0.941 | |  |
| g_Akkermansia | 0.1 (0.2) | 0.2 (0.7) | 0.1 (0.3) | 0.185 | 0.797 | | 0.348 | 0.823 | | 0.098 | 0.808 | |  |
| g_Alistipes | 2.4 (2.0) | 1.9 (2.5) | 2.5 (1.8) | 0.206 | 0.797 | | 0.438 | 0.830 | | 0.114 | 0.808 | |  |
| g_Anaerostipes | 4.2 (2.6) | 4.8 (4.1) | 3.6 (2.5) | 0.479 | 0.872 | | 0.537 | 0.860 | | 0.370 | 0.910 | |  |
| g_Bacteroides | 20.6 (13.3) | 20.8 (14.1) | 20.6 (10.9) | 0.751 | 0.901 | | 0.483 | 0.860 | | 0.863 | 0.941 | |  |
| g_Barnesiella | 0.2 (0.6) | 0.1 (0.3) | 0.6 (1.6) | 0.191 | 0.797 | | 0.335 | 0.823 | | 0.157 | 0.808 | |  |
| g_Bifidobacteria | 13.0 (10.2) | 8.7 (7.7) | 7.8 (6.9) | **0.031** | 0.613 | | **0.013** | 0.487 | | 0.358 | 0.910 | |  |
| g_Bilophilia | 0.0 (0.1) | 0.0 (0.1) | 0.1 (0.1) | 0.347 | 0.851 | | 0.423 | 0.830 | | 0.253 | 0.838 | |  |
| g_Blautia | 8.2 (3.3) | 8.0 (4.8) | 9.4 (4.2) | 0.663 | 0.872 | | 0.677 | 0.871 | | 0.379 | 0.910 | |  |
| g_Butyricicoccus | 0.3 (0.4) | 0.5 (0.4) | 0.5 (0.6) | 0.209 | 0.797 | | 0.291 | 0.823 | | 0.148 | 0.808 | |  |
| g_CAG_56 | 0.2 (0.3) | 0.5 (0.6) | 0.5 (0.7) | 0.183 | 0.797 | | 0.068 | 0.729 | | 0.876 | 0.941 | |  |
| g_Christensenellaceae_R_7_group | 0.5 (1.1) | 0.4 (0.7) | 0.4 (0.5) | 0.968 | 0.972 | | 1 | 1 | | 0.792 | 0.941 | |  |
| g_Clostridium_sensu_stricto_1 | 1.1 (1.8) | 0.6 (0.8) | 0.8 (1.2) | 0.900 | 0.968 | | 0.798 | 0.902 | | 0.738 | 0.941 | |  |
| g_Colidextribacter | 0.1 (0.2) | 0.0 (0.1) | 0.1 (0.2) | 0.550 | 0.872 | | 0.285 | 0.823 | | 0.893 | 0.941 | |  |
| g_Collinsella | 3.5 (4.4) | 2.2 (2.0) | 2.5 (3.4) | 0.659 | 0.872 | | 0.387 | 0.823 | | 0.682 | 0.941 | |  |
| g_Coprobacillus | 0.0 (0.0) | 0.0 (0.1) | 0.0 (0.0) | 0.912 | 0.968 | | 0.889 | 0.927 | | 0.721 | 0.941 | |  |
| g_Coprobacter | 0.1 (0.2) | 0.0 (0.1) | 0.0 (0.1) | 0.552 | 0.872 | | 0.367 | 0.823 | | 0.510 | 0.941 | |  |
| g_Coprococcus | 1.3 (2.1) | 1.4 (2.1) | 1.6 (1.8) | 0.676 | 0.872 | | 0.551 | 0.860 | | 0.532 | 0.941 | |  |
| g_Dialister | 1.8 (3.3) | 2.9 (5.4) | 0.6 (1.6) | 0.178 | 0.797 | | 0.611 | 0.863 | | 0.061 | 0.808 | |  |
| g_Dorea | 3.1 (2.2) | 3.5 (2.8) | 3.9 (2.4) | 0.366 | 0.851 | | 0.204 | 0.783 | | 0.513 | 0.941 | |  |
| g_Eggerthella | 0.1 (0.2) | 0.2 (0.4) | 0.2 (0.4) | 0.119 | 0.797 | | 0.056 | 0.729 | | 0.453 | 0.941 | |  |
| g_Erysipelatoclostridium | 0.3 (0.7) | 0.1 (0.3) | 0.4 (0.7) | 0.688 | 0.872 | | 0.409 | 0.830 | | 0.873 | 0.941 | |  |
| g_Erysipelotrichaceae_UCG_003 | 2.0 (2.3) | 2.6 (2.2) | 1.6 (1.6) | 0.240 | 0.797 | | 0.375 | 0.823 | | 0.160 | 0.808 | |  |
| g_Escheria_Shigella | 0.0 (0.1) | 0.0 (0.0) | 0.0 (0.0) | 0.169 | 0.797 | | 0.289 | 0.823 | | 0.119 | 0.808 | |  |
| g_Faecalibacterium | 4.3 (3.8) | 5.0 (3.6) | 3.5 (2.4) | 0.244 | 0.797 | | 0.796 | 0.902 | | 0.086 | 0.808 | |  |
| g_Faecalitalea | 0.0 (0.0) | 0.1 (0.3) | 0.0 (0.1) | 0.921 | 0.968 | | 0.834 | 0.905 | | 0.744 | 0.941 | |  |
| g_Family_XIII_AD3011_group | 0.2 (0.2) | 0.2 (0.4) | 0.2 (0.2) | 0.317 | 0.851 | | 0.132 | 0.729 | | 0.875 | 0.941 | |  |
| g_Family_XIII_UCG_001 | 0.0 (0.1) | 0.0 (0.1) | 0.0 (0.1) | 0.927 | 0.968 | | 0.802 | 0.902 | | 0.752 | 0.941 | |  |
| g_Flavonifractor | 0.1 (0.1) | 0.1 (0.2) | 0.2 (0.3) | 0.289 | 0.837 | | 0.142 | 0.729 | | 0.614 | 0.941 | |  |
| g_Fusicatenibacter | 1.9 (1.3) | 1.8 (1.4) | 1.9 (1.7) | 0.844 | 0.939 | | 0.609 | 0.863 | | 0.808 | 0.941 | |  |
| g_GCA_900066755 | 0.3 (0.8) | 0.2 (0.4) | 0.2 (0.6) | 0.237 | 0.797 | | 0.797 | 0.902 | | 0.091 | 0.808 | |  |
| g_Gordonibacter | 0.0 (0.0) | 0.0 (0.0) | 0.0 (0.1) | 0.823 | 0.939 | | 0.561 | 0.860 | | 0.915 | 0.941 | |  |
| g_Incertae_Sedis | 0.2 (0.3) | 0.3 (0.4) | 0.4 (0.8) | 0.506 | 0.872 | | 0.287 | 0.823 | | 0.650 | 0.941 | |  |
| g_Intestinibacter | 0.6 (0.5) | 0.6 (0.8) | 0.6 (1.0) | 0.402 | 0.872 | | 0.197 | 0.783 | | 0.657 | 0.941 | |  |
| g_Intestinimonas | 0.0 (0.0) | 0.0 (0.0) | 0.0 (0.1) | 0.699 | 0.872 | | 0.463 | 0.854 | | 0.689 | 0.941 | |  |
| g_Lachnoclostridium | 0.2 (0.2) | 0.1 (0.2) | 0.2 (0.3) | 0.972 | 0.972 | | 0.979 | 0.992 | | 0.832 | 0.941 | |  |
| g_Lachnospira | 0.2 (0.4) | 0.5 (0.7) | 0.2 (0.3) | 0.136 | 0.797 | | 0.095 | 0.729 | | 0.232 | 0.838 | |  |
| g_Lachnospiraceae_FCS020_group | 0.1 (0.3) | 0.1 (0.1) | 0.1 (0.2) | 0.364 | 0.851 | | 0.575 | 0.863 | | 0.168 | 0.808 | |  |
| g_Lachnospiraceae_ND3007_group | 0.2 (0.3) | 0.3 (0.4) | 0.2 (0.3) | 0.348 | 0.851 | | 0.389 | 0.823 | | 0.268 | 0.838 | |  |
| g_Lachnospiraceae_NK4A136_group | 0.4 (0.6) | 0.7 (0.7) | 1.1 (2.1) | 0.059 | 0.706 | | **0.026** | 0.514 | | 0.456 | 0.941 | |  |
| g_Lachnospiraceae_UCG_001 | 0.1 (0.2) | 0.3 (0.4) | 0.2 (0.2) | **0.029** | 0.613 | | 0.135 | 0.729 | | 0.033 | 0.808 | |  |
| g_Lachnospiraceae_UCG_004 | 0.0 (0.1) | 0.0 (0.1) | 0.0 (0.1) | 0.466 | 0.872 | | 0.659 | 0.871 | | 0.233 | 0.838 | |  |
| g_Marvinbryantia | 0.1 (0.1) | 0.1 (0.1) | 0.1 (0.2) | 0.739 | 0.901 | | 0.668 | 0.871 | | 0.539 | 0.941 | |  |
| g_Megamonas | 0.5 (2.1) | 0.0 (0.0) | 1.0 (2.9) | 0.702 | 0.872 | | 0.765 | 0.902 | | 0.437 | 0.941 | |  |
| g_Monoglobus | 0.9 (0.8) | 0.9 (0.9) | 1.1 (1.0) | 0.606 | 0.872 | | 0.335 | 0.823 | | 0.808 | 0.941 | |  |
| g_NK4A214_group | 0.1 (0.2) | 0.3 (0.6) | 0.2 (0.4) | 0.404 | 0.872 | | 0.182 | 0.783 | | 0.923 | 0.941 | |  |
| g_Odoribacter | 0.2 (0.2) | 0.2 (0.2) | 0.2 (0.2) | 0.580 | 0.872 | | 0.308 | 0.823 | | 0.863 | 0.941 | |  |
| g_Oscillibacter | 0.1 (0.2) | 0.1 (0.1) | 0.1 (0.2) | 0.102 | 0.797 | | 0.110 | 0.729 | | 0.101 | 0.808 | |  |
| g_Oscillospira | 0.0 (0.0) | 0.0 (0.0) | 0.0 (0.1) | 0.556 | 0.872 | | 0.523 | 0.860 | | 0.425 | 0.941 | |  |
| g_Oxalobacter | 0.0 (0.1) | 0.0 (0.0) | 0.0 (0.1) | 0.590 | 0.872 | | 0.322 | 0.823 | | 0.824 | 0.941 | |  |
| g_Parabacteroides | 1.3 (1.1) | 1.0 (1.5) | 1.4 (1.6) | 0.217 | 0.797 | | 0.111 | 0.729 | | 0.567 | 0.941 | |  |
| g_Paraprevotella | 0.1 (0.3) | 0.1 (0.2) | 0.2 (0.3) | 0.685 | 0.872 | | 0.596 | 0.863 | | 0.522 | 0.941 | |  |
| g_Parasutterella | 0.3 (1.2) | 0.2 (0.4) | 0.1 (0.2) | 0.506 | 0.872 | | 0.524 | 0.860 | | 0.360 | 0.910 | |  |
| g_Phascolarctobacterium | 0.3 (0.4) | 0.5 (0.8) | 0.9 (1.0) | **0.038** | 0.613 | | **0.029** | 0.514 | | 0.187 | 0.821 | |  |
| g_Prevotella | 0.9 (2.2) | 1.4 (3.3) | 0.7 (1.9) | 0.766 | 0.904 | | 0.822 | 0.905 | | 0.502 | 0.941 | |  |
| g_Romboutsia | 1.0 (1.3) | 1.9 (4.1) | 2.2 (2.7) | 0.656 | 0.872 | | 0.430 | 0.830 | | 0.607 | 0.941 | |  |
| g_Roseburia | 2.3 (3.5) | 2.1 (1.8) | 1.3 (1.1) | 0.291 | 0.837 | | 0.933 | 0.959 | | 0.122 | 0.808 | |  |
| g_Ruminococcus | 3.8 (4.3) | 3.3 (3.9) | 4.6 (4.7) | 0.593 | 0.872 | | 0.728 | 0.902 | | 0.339 | 0.910 | |  |
| g_Streptococcus | 0.9 (1.1) | 1.1 (1.7) | 1.4 (3.9) | 0.536 | 0.872 | | 0.278 | 0.823 | | 0.876 | 0.941 | |  |
| g_Subdoligranulum | 3.6 (3.1) | 2.9 (2.7) | 3.1 (2.9) | 0.472 | 0.872 | | 0.223 | 0.803 | | 0.972 | 0.972 | |  |
| g_Sutterella | 0.2 (0.4) | 0.2 (0.4) | 0.2 (0.3) | 0.416 | 0.872 | | 0.783 | 0.902 | | 0.219 | 0.838 | |  |
| g_Terrisporobacter | 0.1 (0.2) | 0.0 (0.1) | 0.0 (0.1) | 0.614 | 0.872 | | 0.636 | 0.864 | | 0.327 | 0.910 | |  |
| g_Tuzzerella | 0.1 (0.1) | 0.0 (0.1) | 0.1 (0.1) | **0.020** | 0.613 | | 0.113 | 0.729 | | 0.026 | 0.808 | |  |
| g_Tyzzerella | 0.1 (0.3) | 0.1 (0.3) | 0.0 (0.1) | **0.043** | 0.613 | | **0.014** | 0.487 | | 0.679 | 0.941 | |  |
| g_UBA1819 | 0.1 (0.1) | 0.1 (0.1) | 0.2 (0.4) | 0.287 | 0.837 | | 0.529 | 0.860 | | 0.151 | 0.808 | |  |
| g_UCG_002 | 0.6 (0.7) | 0.6 (1.0) | 1.1 (1.5) | 0.585 | 0.872 | | 0.632 | 0.864 | | 0.355 | 0.910 | |  |
| g_UCG_003 | 0.1 (0.2) | 0.3 (0.5) | 0.2 (0.3) | 0.213 | 0.797 | | 0.098 | 0.729 | | 0.643 | 0.941 | |  |
| g_UCG_005 | 0.3 (0.4) | 0.3 (0.6) | 0.4 (0.5) | 0.438 | 0.872 | | 0.843 | 0.905 | | 0.194 | 0.821 | |  |
| g_UCG_009 | 0.0 (0.0) | 0.0 (0.0) | 0.0 (0.0) | 0.962 | 0.972 | | 0.873 | 0.924 | | 0.756 | 0.941 | |  |

All values are mean (SD); n, number of participants with available data;^1^*p*-values are the result Kruskal-Wallis test; ^2^*p*-values are the result of Mann-Whitney test; *q*-values are the result of correction of *p*-values for multiple testing (Benjamini-Hochberg FDR); Intention to treat population was modified to include only those who provided sufficient sample for analysis at baseline and end of the intervention.

Modified ITT (n = 79), control = 25, whole almond = 29, ground almond = 25

**SUPPLEMENTARY TABLE 2 Table of volatile organic compounds significantly increased by almond consumption (pooled whole and ground) in comparison to control in the modified intention to treat population**

|  | **Higher following** | ***p*-value** | ***q*-value** |
| --- | --- | --- | --- |
| **2-methylbutanoic acid** | Almonds | **0.004** | 0.057 |
| **3-methylbutanoic acid** | Almonds | **0.006** | 0.057 |
| **Nonanal** | Almonds | **0.007** | 0.057 |

Modified ITT (n = 76), control = 22, pooled almond = 54; *p-*values are the result of *t*-tests; *q*-values are the result of correction of *p-*values for multiple testing (Benjamini-Hochberg FDR).

**SUPPLEMENTARY TABLE 3 Incidence of gastrointestinal symptoms in the intention to treat population**

|  | **Control muffin** | | **Whole almond** | | **Ground almond** | | ***p*-value** |
| --- | --- | --- | --- | --- | --- | --- | --- |
|  | Baseline  (n=26) | Endpoint  (n=25) | Baseline  (n=32) | Endpoint  (n=30) | Baseline  (n=28) | Endpoint  (n=26) |  |
| **Pain/discomfort** | 0.6 (1.1) | 0.6 (1.5) | 1.2 (1.5) | 1.3 (1.8) | 0.8 (1.4) | 0.9 (1.3) | 0.125 |
| **Heartburn** | 0.2 (0.6) | 0.3 (0.7) | 0.2 (0.5) | 0.3 (1.2) | 0.1 (0.6) | 0.3 (1.7) | 0.826 |
| **Reflux** | 0.0 (0.0) | 0.2 (0.6) | 0.2 (1.2) | 0.3 (0.9) | 0.1 (0.4) | 0.2 (0.4) | 0.782 |
| **Nausea** | 0.4 (0.8) | 0.6 (1.6) | 0.7 (1.3) | 0.5 (1.2) | 0.5 (1.1) | 0.7 (1.5) | 0.717 |
| **Abdominal gurgling** | 1.2 (1.8) | 1.7 (2.2) | 1.3 (1.7) | 1.5 (2.2) | 1.0 (1.7) | 1.3 (2.1) | 0.820 |
| **Bloating** | 1.5 (1.8) | 0.8 (1.7) | 1.6 (2.2) | 1.8 (2.3) | 1.3 (2.0) | 1.1 (1.9) | 0.120 |
| **Belching** | 1.2 (1.7) | 1.2 (1.8) | 1.2 (2.0) | 1.3 (2.2) | 1.3 (2.0) | 1.2 (2.0) | 0.928 |
| **Flatulence** | 3.9 (2.5) | 1.9 (2.6) | 2.9 (2.9) | 2.5 (2.8) | 3.4 (2.9) | 2.7 (2.9) | 0.836 |
| **Constipation** | 0.8 (1.4) | 0.3 (0.7) | 0.4 (0.9) | 0.3 (0.7) | 0.4 (1.1) | 0.4 (0.8) | 0.681 |
| **Diarrhea** | 0.0 (0.2) | 0.1 (0.3) | 0.2 (0.4) | 0.2 (0.5) | 0.1 (0.4) | 0.0 (0.2) | 0.130 |
| **Loose stools** | 0.6 (1.0) | 0.5 (0.8) | 0.6 (0.9) | 1.0 (1.6) | 0.8 (1.4) | 0.4 (1.0) | 0.267 |
| **Hard stools** | 1.2 (1.5) | 0.6 (1.1) | 0.5 (0.8) | 0.4 (0.9) | 0.9 (1.5) | 0.2 (0.6) | 0.326 |
| **Urgency** | 0.3 (0.5) | 0.3 (0.9) | 0.6 (1.2) | 0.7 (1.4) | 0.5 (1.1) | 0.2 (0.6) | 0.500 |
| **Incomplete evacuation** | 1.0 (1.7) | 0.4 (0.7) | 0.5 (1.1) | 0.5 (1.1) | 1.0 (1.7) | 0.6 (1.5) | 0.970 |
| **Tiredness** | 2.6 (2.3) | 2.6 (2.8) | 2.4 (2.4) | 2.4 (2.6) | 1.9 (2.0) | 1.4 (1.7) | 0.293 |
| **Overall symptoms** | 1.7 (2.1) | 1.6 (2.0) | 1.5 (2.0) | 1.5 (2.1) | 1.6 (2.1) | 1.5 (2.2) | 0.890 |

All values are mean days/week on which that symptom was experienced (SD); n, number of participants with available data; *P*-values are the result of Kruskal-Wallis test on endpoint values.

Numbers in each group: ITT (n = 87), control = 26, whole almond = 33, ground almond = 28

**SUPPLEMENTARY TABLE 4 Severity of gastrointestinal symptoms in the intention to treat population**

|  | **Control muffin** | | **Whole almond** | | **Ground almond** | | ***p*-value** |
| --- | --- | --- | --- | --- | --- | --- | --- |
|  | Baseline  (n = 26) | Endpoint  (n = 25) | Baseline  (n = 32) | Endpoint  (n = 30) | Baseline  (n = 28) | Endpoint  (n =26) |  |
| **Pain/discomfort** | 0.1 (0.2) | 0.1 (0.3) | 0.2 (0.3) | 0.3 (0.4) | 0.2 (0.3) | 0.1 (0.2) | 0.093 |
| **Heartburn** | 0.0 (0.1) | 0.0 (0.1) | 0.0 (0.1) | 0.1 (0.2) | 0.0 (0.1) | 0.0 (0.1) | 0.819 |
| **Reflux** | 0.0 (0.0) | 0.0 (0.1) | 0.0 (0.2) | 0.0 (0.2) | 0.0 (0.1) | 0.0 (0.1) | 0.782 |
| **Nausea** | 0.1 (0.1) | 0.1 (0.3) | 0.1 (0.2) | 0.1 (0.2) | 0.1 (0.2) | 0.1 (0.2) | 0.708 |
| **Abdominal gurgling** | 0.2 (0.3) | 0.3 (0.4) | 0.2 (0.3) | 0.2 (0.3) | 0.2 (0.3) | 0.2 (0.4) | 0.786 |
| **Bloating** | 0.3 (0.3) | 0.2 (0.4) | 0.3 (0.4) | 0.3 (0.4) | 0.2 (0.4) | 0.2 (0.3) | 0.121 |
| **Belching** | 0.2 (0.2) | 0.2 (0.3) | 0.2 (0.3) | 0.2 (0.3) | 0.2 (0.3) | 0.2 (0.3) | 0.932 |
| **Flatulence** | 0.6 (0.4) | 0.5 (0.4) | 0.5 (0.5) | 0.4 (0.5) | 0.6 (0.5) | 0.4 (0.4) | 0.886 |
| **Constipation** | 0.1 (0.2) | 0.0 (0.1) | 0.1 (0.2) | 0.0 (0.1) | 0.1 (0.2) | 0.1 (0.1) | 0.694 |
| **Diarrhea** | 0.0 (0.0) | 0.0 (0.1) | 0.0 (0.1) | 0.1 (0.1) | 0.0 (0.1) | 0.0 (0.1) | 0.135 |
| **Loose stools** | 0.1 (0.2) | 0.1 (0.2) | 0.1 (0.2) | 0.2 (0.3) | 0.1 (0.2) | 0.1 (0.1) | 0.233 |
| **Hard stools** | 0.2 (0.2) | 0.1 (0.2) | 0.1 (0.1) | 0.1 (0.1) | 0.2 (0.3) | 0.0 (0.1) | 0.332 |
| **Urgency** | 0.0 (0.1) | 0.1 (0.1) | 0.1 (0.3) | 0.1 (0.2) | 0.1 (0.3) | 0.0 (0.1) | 0.446 |
| **Incomplete evacuation** | 0.2 (0.3) | 0.1 (0.1) | 0.1 (0.2) | 0.1 (0.2) | 0.2 (0.3) | 0.1 (0.3) | 0.977 |
| **Tiredness** | 0.5 (0.6) | 0.5 (0.6) | 0.5 (0.5) | 0.4 (0.5) | 0.4 (0.5) | 0.3 (0.4) | 0.302 |
| **Overall symptoms** | 0.2 (0.3) | 0.2 (0.3) | 0.2 (0.3) | 0.2 (0.3) | 0.2 (0.3) | 0.2 (0.4) | 0.899 |

Severity was rated daily on a scale as follows: 0, absent; 1, mild; 2, moderate; 3, severe; All values are mean severity over the number of days recorded (SD); n, number of participants with available data; *P*-values are the result of Kruskal-Wallis test on endpoint values.

Numbers in each group: ITT (n = 87), control = 26, whole almond = 33, ground almond = 28.

**SUPPLEMENTARY TABLE 5 Energy and nutrient intakes at baseline and endpoint in the intention to treat population**

|  | **Control muffin** | | **Whole almond** | | **Ground almond** | | **ANCOVA** | **Significant comparisons** |
| --- | --- | --- | --- | --- | --- | --- | --- | --- |
|  | Baseline  (n = 26) | Endpoint  (n = 25) | Baseline  (n = 33) | Endpoint  (n = 30) | Baseline  (n = 28) | Endpoint  (n = 26) | *p*-value | *p*-value^1^ |
| **Energy**  kcal/d | 1942.3 (479.2) | 1757.8 (447.2) | 1956.7 (482.4) | 2031.7 (477.9) | 1922.2 (448.0) | 1911.9 (500.6) | **0.048** | Whole vs. control = 0.042 |
| **Protein**  g/d | 74.6 (21.1) | 68.1 (17.0) | 72.7 (19.6) | 79.4 (19.7) | 79.3 (20.4) | 79.9 (24.4) | **0.021** | Whole vs. control = 0.018 |
| %E | 15.6 (3.6) | 15.7 (2.8) | 15.1 (3.2) | 15.8 (2.8) | 16.8 (4.5) | 17.0 (4.3) | 0.588 | - |
| **Carbohydrate**  g/d | 218.7 (63.8) | 192.9 (54.7) | 214.6 (66.6) | 198.7 (58.8) | 211.7 (63.1) | 189.9 (65.1) | 0.834 | - |
| %E | 45.3 (8.0) | 44.1 (6.1) | 43.7 (7.5) | 39.0 (7.6) | 44.1 (7.3) | 39.5 (7.9) | **0.014** | Whole vs. control = 0.018 |
| **Fat**  g/d | 79.1 (24.8) | 73.7 (21.3) | 84.9 (26.1) | 93.9 (27.5) | 78.2 (20.0) | 87.0 (24.3) | **0.012** | Whole vs. control = 0.012 |
| %E | 36.3 (5.6) | 37.6 (4.0) | 39.0 (6.7) | 41.6 (6.7) | 36.5 (4.4) | 41.1 (6.4) | 0.052 | - |
| **Saturated fat**  g/d | 27.6 (11.2) | 26.5 (8.7) | 28.5 (8.5) | 24.9 (8.1) | 27.2 (9.3) | 22.4 (8.6) | 0.124 | - |
| %E | 12.5 (3.5) | 13.5 (2.8) | 13.2 (3.0) | 11.0 (2.4) | 12.5 (2.4) | 10.4 (3.3) | **<0.001** | Whole vs. control = 0.001  Ground vs. control < 0.001 |
| **MUFA**  g/d | 19.2 (7.2) | 22.3 (6.1) | 22.8 (12.5) | 34.7 (9.4) | 21.5 (7.1) | 36.1 (10.7) | **<0.001** | Whole vs. control <0.001  Ground vs. control <0.001 |
| %E | 9.1 (3.0) | 11.7 (2.9) | 10.4 (4.5) | 15.6 (3.3) | 10.1 (3.0) | 17.2 (4.0) | **<0.001** | Whole vs. control <0.001  Ground vs. control <0.001 |
| **PUFA**  g/d | 9.4 (4.4) | 11.3 (3.8) | 11.3 (6.7) | 14.3 (4.3) | 10.0 (3.6) | 13.7 (4.5) | 0.065 | - |
| %E | 4.4 (2.0) | 5.9 (1.8) | 5.1 (2.4) | 6.4 (1.5) | 4.8 (1.7) | 6.5 (1.4) | 0.458 | - |
| **Sugars**  g/d | 90.0 (36.9) | 79.6 (26.3) | 88.1 (37.3) | 74.7 (31.0) | 93.2 (45.1) | 70.4 (34.5) | 0.209 | - |
| %E | 18.4 (5.2) | 18.1 (3.6) | 17.8 (4.9) | 14.6 (4.9) | 19.3 (7.9) | 14.8 (5.0) | **< 0.001** | Whole vs. control = 0.002  Ground vs. control <0.001 |
| **Starch**  g/d | 126.5 (36.3) | 111.1 (33.5) | 124.5 (43.0) | 122.0 (39.7) | 116.8 (30.8) | 117.4 (39.8) | 0.208 | - |
| %E | 26.4 (5.9) | 25.4 (4.7) | 25.4 (6.4) | 24.0 (6.1) | 24.4 (5.0) | 24.5 (6.5) | 0.471 | - |
| **Fiber,** g/d | 20.9 (7.8) | 17.5 (5.9) | 21.9 (8.4) | 26.8 (10.2) | 19.2 (6.7) | 24.0 (6.8) | **<0.001** | Whole vs. control <0.001  Ground vs. control <0.001 |
| **NSP,** g/d | 15.2 (6.2) | 12.5 (4.5) | 16.1 (7.5) | 20.0 (9.4) | 14.0 (5.1) | 17.6 (5.7) | **<0.001** | Whole vs. control <0.001  Ground vs. control <0.001 |
| **Sodium,** mg/d | 2084.1 (543.8) | 2208.5 (550.0) | 2139.6 (611.9) | 2131.2 (737.1) | 2145.9 (747.9) | 2546.3 (813.5) | 0.056 | - |
| **Potassium,** mg/d | 2274.8 (566.4) | 2058.4 (576.8) | 2281.1 (886.6) | 2610.8 (942.9) | 2440.8 (664.3) | 2470.5 (754.6) | **0.012** | Whole vs. control = 0.009 |
| **Calcium,** mg/d | 600.1 (204.1) | 598.5 (203.3) | 614.5 (221.2) | 722.0 (213.8) | 649.0 (254.3) | 711.4 (245.0) | 0.075 | - |
| **Magnesium,** mg/d | 230.6 (66.7) | 199.4 (61.9) | 247.5 (95.5) | 373.0 (79.8) | 250.7 (77.7) | 343.1 (108.9) | **<0.001** | Whole vs. control <0.001  Ground vs. control <0.001 |
| **Phosphorus,** mg/d | 934.3 (286.7) | 989.2 (252.9) | 934.8 (301.7) | 1153.0 (284.4) | 1012.4 (254.2) | 1144.6 (309.0) | **0.043** | Whole vs. control  = 0.040 |
| **Iron,** mg/d | 9.3 (2.9) | 9.1 (4.8) | 9.0 (2.5) | 10.8 (3.3) | 9.7 (3.0) | 9.9 (4.2) | 0.120 | - |
| **Copper,** mg/d | 1.3 (0.4) | 2.3 (5.2) | 2.2 (4.9) | 1.8 (0.5) | 1.4 (0.4) | 1.8 90.7) | 0.784 | - |
| **Chloride,** mg/d | 3006.6 (821.5) | 3183.4 (879.7) | 3082.8 (953.8) | 2960.8 (1112.2) | 3123.6 (1045.6) | 3843.0 (1105.9) | **0.006** | Whole vs. ground  = 0.005 |
| **Zinc,** mg/d | 6.3 (1.9) | 6.2 (2.3) | 6.8 (3.1) | 8.1 (1.8) | 7.4 (2.6) | 7.5 (2.4) | **0.006** | Whole vs. control=0.004 |
| **Manganese,** mg/d | 3.3 (3.0) | 2.3 (0.8) | 6.6 (17.7) | 3.8 (1.1) | 3.2 (1.9) | 3.6 (1.5) | **<0.001** | Whole vs control <0.001  Ground vs. control = 0.001 |
| **Selenium,** μg/d | 35.1 (18.6) | 39.1 (17.1) | 33.0 (14.2) | 37.6 (15.1) | 45.1 (17.8) | 37.8 (14.9) | 0.538 | - |
| **Water,** g/d | 1498.3 (836.0) | 1551.7 (1022.3) | 1711.8 (862.1) | 1614.7 (698.8) | 1378.5 (606.9) | 1699.5 (829.5) | 0.239 | - |

All values are mean (SD); MUFA, monounsaturated fatty acids; PUFA, polyunsaturated fatty acids; NSP, non-starch polysaccharides; g, grams; %E, percentage of total energy; mg, miligrams; μg, micrograms; *p*-values are the result of an ANCOVA with baseline values as a covariate. ^1^*p*-values are the result of a Bonferroni *post hoc* test.

**SUPPLEMENTARY TABLE 6 Body weight and body composition at baseline and endpoint in the intention to treat population**

|  | **Control muffin** | | **Whole almond** | | **Ground almond** | | ***p*-value** |
| --- | --- | --- | --- | --- | --- | --- | --- |
|  | Baseline  (n = 26) | Endpoint  (n = 25) | Baseline  (n = 33) | Endpoint  (n = 30) | Baseline  (n = 28) | Endpoint  (n = 26) |  |
| **Body weight (kg)** | 65.0 (8.1) | 65.2 (7.7) | 64.4 (11.9) | 64.5 (11.9) | 62.4 (9.6) | 63.1 (9.9) | 0.180 |
| **Body mass index (kg/m^2^)** | 23.6 (2.7) | 23.7 (2.6) | 22.6 (2.9) | 22.6 (3.0) | 22.7 (2.9) | 22.9 (3.0) | 0.194 |
| **Fat (%)** | 29.9 (6.0) | 30.1 (5.8) | 26.9 (6.7) | 26.3 (7.3) | 27.2 (8.2) | 27.7 (8.7) | 0.494 |
| **Fat mass (kg)** | 19.7 (5.7) | 19.9 (5.7) | 17.4 (6.5) | 17.1 (6.5) | 17.4 (7.4) | 17.9 (7.6) | 0.802 |
| **Fat free mass (kg)** | 45.3 (4.5) | 45.3 (3.8) | 45.6 (6.2) | 47.4 (9.7) | 45.0 (5.8) | 45.2 (6.5) | 0.230 |

All values are mean (SD); *p*-values are the result of an ANCOVA with baseline values as a covariate.

**SUPPLEMENTARY TABLE 7 Quality of life in the intention to treat population**

|  | **Control muffin** | | | | **Whole almond** | | | | **Ground almond** | | | | ***p*-value** |
| --- | --- | --- | --- | --- | --- | --- | --- | --- | --- | --- | --- | --- | --- |
|  | n | Baseline | n | Endpoint | n | Baseline | n | Endpoint | n | Baseline | n | Endpoint |  |
| **Physical functioning** | 25 | 93.0 (17.0) | 25 | 97.2 (7.2) | 33 | 95.2 (10.6) | 30 | 95.0 (10.0) | 27 | 95.9 (13.5) | 26 | 98.8 (2.1) | 0.303 |
| **Role limitations due to physical health** | 25 | 98.0 (6.9) | 25 | 92.0 (20.1) | 33 | 91.7 (23.9) | 30 | 91.7 21.1) | 28 | 97.3 (10.4) | 26 | 97.1 (10.8) | 0.483 |
| **Role limitations due to emotional problems** | 25 | 80.0 (30.4) | 25 | 85.3 (32.0) | 33 | 74.7 (37.3) | 30 | 84.4 (31.2) | 28 | 79.8 (37.8) | 26 | 91.0 (22.2) | 0.727 |
| **Energy/fatigue** | 24 | 58.1 (21.3) | 25 | 57.5 (20.6) | 33 | 60.6 (17.0) | 30 | 60.3 (17.1) | 28 | 63.5 (14.4) | 26 | 64.5 (14.3) | 0.478 |
| **Emotional wellbeing** | 24 | 75.0 (16.5) | 25 | 75.5 (16.6) | 33 | 75.9 (14.3) | 30 | 77.9 (14.2) | 28 | 76.6 (12.2) | 26 | 79.8 (10.0) | 0.808 |
| **Social functioning** | 25 | 87.0 (24.1) | 25 | 93.0 (16.6) | 33 | 83.3 (19.7) | 30 | 88.8 (16.9) | 28 | 90.2 (17.5) | 26 | 94.2 (12.4) | 0.278 |
| **Pain** | 24 | 89.2 (16.3) | 25 | 92.1 (10.2) | 33 | 87.2 (16.2) | 30 | 85.3 (20.0) | 27 | 89.1 (13.6) | 26 | 88.8 (12.8) | 0.560 |
| **General health** | 25 | 73.2 (20.8) | 25 | 72.6 (18.5) | 33 | 72.0 (15.1) | 30 | 75.8 (13.5) | 28 | 75.0 (17.0) | 26 | 77.3 (16.3) | 0.619 |

All values are mean quality of life score (SD); n, number of participants with available data; *p*-values are the result of Kruskal-Wallis test on endpoint values.

Numbers in each group: ITT (n = 87), control = 26, whole almond = 33, ground almond = 28

**Legend for supplementary figure**

**SUPPLEMENTARY FIGURE 1 Summary of the study design, interventions and procedures of a randomized controlled trial investigating the impact of almonds (whole or ground) on gut microbiology, physiology and function in 87 healthy adults**

Participants used the 7-day diary to record stool frequency and consistency, gastrointestinal symptoms and dietary intake at baseline and endpoint. During the intervention period participants were contacted via phone for the purposes of compliance checks and troubleshooting. *A subgroup of participants (n = 48) took part in measurement of gut transit, pH and pressure.

SCFA, short chain fatty acid; VOC, volatile organic compound.
