# Supplementary figures and images for: The impact of almonds and almond processing on gastrointestinal physiology, luminal microbiology, and gastrointestinal symptoms: a randomized controlled trial and mastication study
Source: Am J Clin Nutr. 2022 Sep 20;116(6):1790–804. doi: 10.1093/ajcn/nqac265 (PMC9761756; doi:10.1093/ajcn/nqac265)

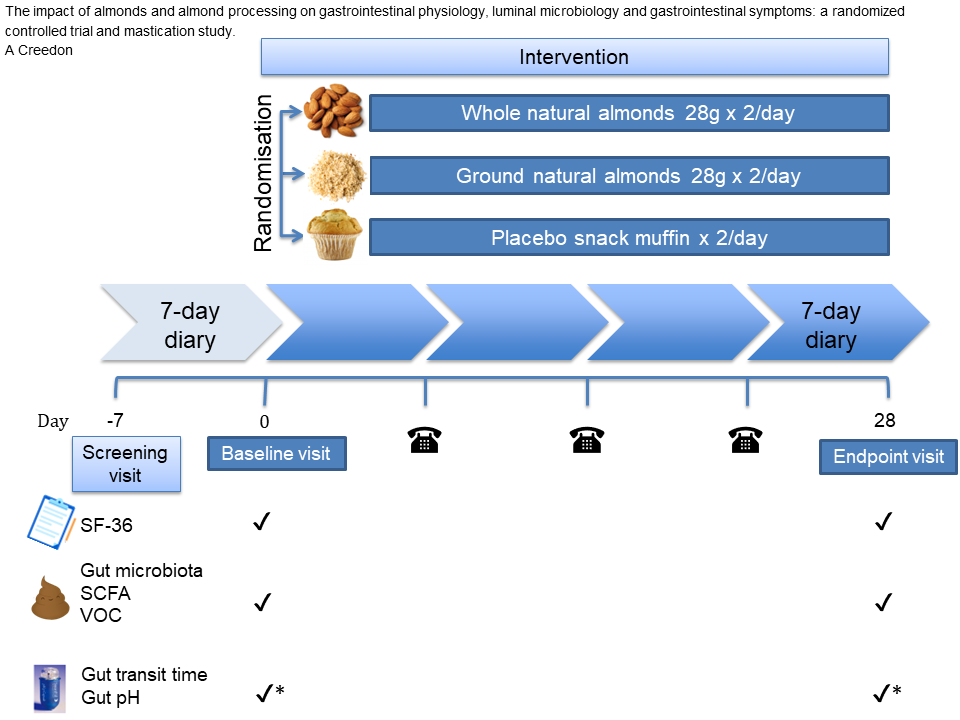

Supplement: nqac265_Supplemental_Files [file nqac265_supplemental_files.zip › Supplementary figure 1_online_colour_header.png]
